# Combinatorial pathway balancing provides biosynthetic access to 2-fluoro-*cis*,*cis*-muconate in engineered *Pseudomonas putida*

**DOI:** 10.1016/j.checat.2021.09.002

**Published:** 2021-11-18

**Authors:** Nicolas T. Wirth, Pablo I. Nikel

**Affiliations:** 1 The Novo Nordisk Foundation Center for Biosustainability, Technical University of Denmark, 2800 Kgs. Lyngby, Denmark

**Keywords:** *Pseudomonas putida*, metabolic engineering, synthetic biology, 2-fluoro-*cis*,*cis*-muconate, new-to-industry product, biocatalysis, *ortho*-cleavage, building blocks, pathway balancing, expression systems

## Abstract

The wealth of bio-based building blocks produced by engineered microorganisms seldom include halogen atoms. Muconate is a platform chemical with a number of industrial applications that could be broadened by introducing fluorine atoms to tune its physicochemical properties. The soil bacterium *Pseudomonas putida* naturally assimilates benzoate via the *ortho*-cleavage pathway with *cis*,*cis*-muconate as intermediate. Here, we harnessed the native enzymatic machinery (encoded within the *ben* and *cat* gene clusters) to provide catalytic access to 2-fluoro-*cis*,*cis*-muconate (2-FMA) from fluorinated benzoates. The reactions in this pathway are highly imbalanced, leading to accumulation of toxic intermediates and limited substrate conversion. By disentangling regulatory patterns of *ben* and *cat* in response to fluorinated effectors, metabolic activities were adjusted to favor 2-FMA biosynthesis. After implementing this combinatorial approach, engineered *P*. *putida* converted 3-fluorobenzoate to 2-FMA at the maximum theoretical yield. Hence, this study illustrates how synthetic biology can expand the diversity of nature's biochemical catalysis.

## Highlights

• *Pseudomonas putida* processes fluorinated benzoates via the *ortho*-cleavage pathway
• Imbalanced utilization of fluorinated substrates by *P*. *putida* results in toxic effects
• Pathway modules show divergent transcriptional responses to fluorinated metabolites
• Balancing enzyme activities enables efficient 2-fluoro-*cis*,*cis*-muconate production

## The bigger picture

Even after decades of research, the chemical nature and structural diversity of building blocks obtained by biocatalysis is restricted to a handful of compounds. Moreover, the potential of bio-based addition of halogen atoms is yet to be fully exploited. Here, 2-fluoro-*cis*,*cis*-muconate (2-FMA) was targeted as a new-to-industry chemical. 2-FMA acts as precursor of complex organic structures by its two terminal carboxyl groups, providing access to novel fluorinated polymers and bioactives for medicine and agriculture. Virtually impossible to produce via traditional chemical synthesis, 2-FMA was obtained by leveraging the rich metabolism of the platform bacterium *Pseudomonas putida*. Efficient conversion of 3-fluorobenzoate, a low-cost haloarene, into 2-FMA is only possible through finely orchestrated biochemistry. Such balance was achieved by combinatorial substitution of native regulatory elements—a strategy that can be also implemented for biosynthesis of other value-added molecules.

## Graphical abstract

**Figure:**
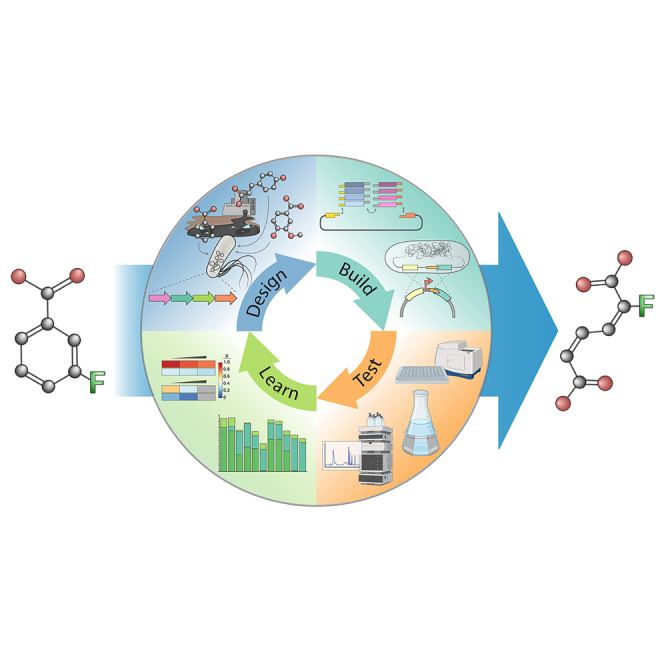

## Teaser

Biocatalysis can provide access to value-added fluorinated compounds that are difficult to synthesize chemically. In this article, Wirth and Nikel describe a strategy for biological production of 2-fluoro-*cis*,*cis*-muconate, a fluorinated derivative of muconate (classified as a top-50 platform molecule). *Pseudomonas* strains were reprogrammed through a rational metabolic engineering approach applied to native pathways for biodegradation of aromatic xenobiotics. Following this blueprint, the catalytic potential of microbial biochemical pathways can be harnessed toward expanding the spectrum of fluorinated building blocks available for industrial applications.

## Introduction

*cis*,*cis*-Muconate [(2*Z*,4*Z*)-2,4-hexadienedioic acid; *cc*MA] is a value-added product with conjugated double bonds and reactive dicarboxylic groups, which facilitate the use of this building block in a large variety of reactions toward both commodity and specialty chemicals.^1^, ^2^, ^3^, ^4^, ^5^, ^6^ The configuration of its functional groups makes *cc*MA particularly suitable for polymerization reactions that yield synthetic resins and biodegradable polymers.^2^ The list of compounds accessible through *cc*MA includes many commercially important chemicals, e.g., adipic acid (a top-50 bulk chemical),^7^ caprolactam, and terephthalic and trimellitic acids. These molecules find a variety of manufacturing uses in the form of nylon-6,6, polytrimethylene terephthalate, polyethylene terephthalate, dimethyl terephthalate, trimellitic anhydride, industrial plastics, polyester, food ingredients, pharmaceuticals, plasticizers, and cosmetics.^3^ Over the past decades, efforts have focused on replacing oil-based processes for *cc*MA production with biotechnological alternatives.^7^, ^8^, ^9^, ^10^, ^11^, ^12^, ^13^, ^14^, ^15^ These approaches largely fall into two categories: (1) *de novo* production of aromatic precursors from sugars or glycerol via the shikimate pathway, followed by conversion to catechol and ring-cleavage to *cc*MA, or (2) direct bioconversion of aromatic feedstocks.^16^, ^17^, ^18^

Although such bio-based methods for *cc*MA synthesis are available, the molecule's structural scope is still severely restricted and can only be expanded by post-production modifications. The intrinsic value of bio-based *cc*MA production could be multiplied by introducing halogen atoms into the molecule and its derivatives, enabling access to products that can hardly be synthesized chemically^19^—most of which are new to industry. Replacing a hydrogen atom with fluorine (F) has become an essential manipulation in organic chemistry,^20^ and the presence of even a single F atom significantly enhances chemical properties of drugs and building blocks.^21^, ^22^, ^23^ The effect of fluorination on *cc*MA-derived products is of particular interest for polymer applications. Introducing F into a polymer structure brings about a suite of industrially relevant features, e.g., inertness to acids, bases, solvents, and oils; low dielectric constant; low refractive index; high resistance to aging and oxidation; and low surface tension.^24^ Since the targeted introduction of F into a complex organic structure is difficult to achieve through chemical synthesis, emergent strategies toward the synthesis of fluorinated building blocks are urgently needed.^21^^,^^25^ Bio-based solutions for the production of such building blocks are particularly promising,^26^^,^^27^ since traditional approaches to fluorination involve highly reactive, often unspecific reagents and are insensitive to the stereochemistry of the product.^28^

Against this background, this study describes a bio-based strategy to produce fluorinated *cis*,*cis*-muconate via whole-cell bioconversion with engineered *Pseudomonas* cells. *Pseudomonas putida* is a soil bacterium that became a platform for the production of value-added compounds owing to its remarkably versatile metabolism and high levels of stress resistance.^29^, ^30^, ^31^, ^32^ Inspired by the biochemical wiring in a natural chlorobenzoate-degrading *Pseudomonas* species (*Pseudomonas knackmussii*), we upgraded the catalytic landscape of *P*. *putida* to efficiently convert fluorinated benzoates into the corresponding halogenated muconate. The biocatalyst was further optimized on several iterations of the “design-build-test-learn cycle” of synthetic metabolic engineering.^33^ Adopting a combinatorial pathway-balancing approach ultimately enabled the complete conversion of 3-fluorobenzoate (3-FBz) into isomerically pure 2-fluoro-*cis*,*cis*-muconate [(2*E*,4*Z*)-2-fluorohexa-2,4-dienedioate; 2-FMA] at the maximum theoretical yield.

## Results

### A comparative genome analysis of *P. putida* and *P*. *knackmussii* unveils the enzymatic repertoire involved in fluoroaromatic metabolism

**Figure 1:**
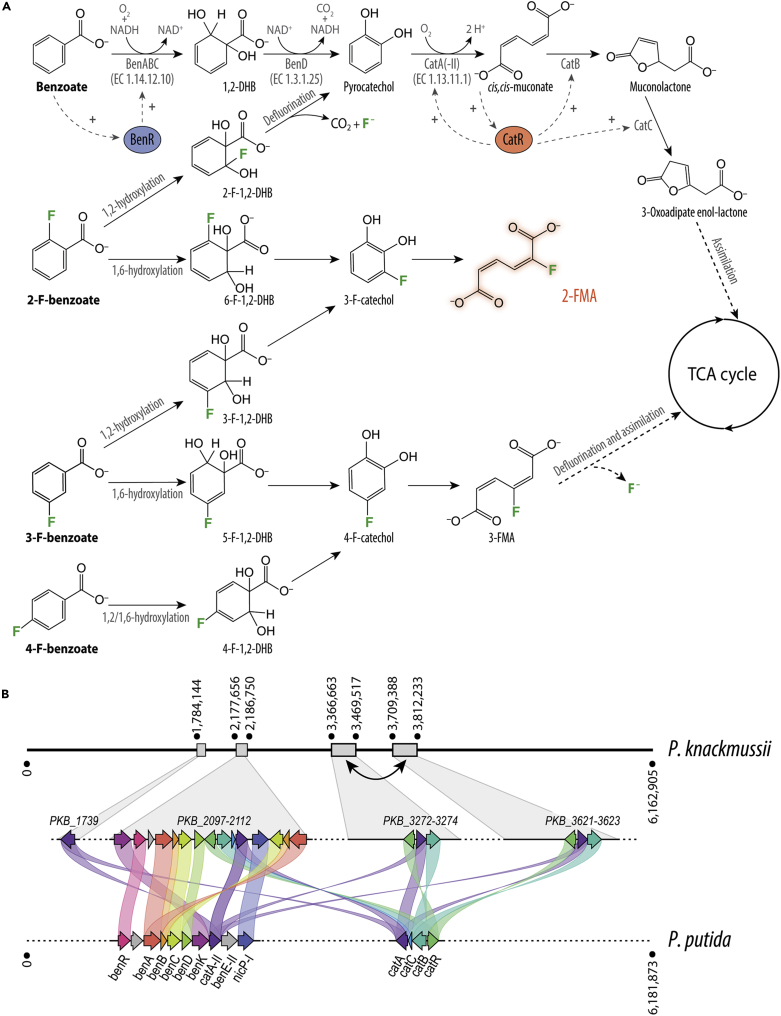
(Fluoro)benzoate degradation in wild-type *Pseudomonas* species (A) Biochemical activities acting on benzoate and its fluorine (F)-substituted derivatives 2-FBz, 3-FBz, and 4-FBz. Positive transcriptional regulation by BenR and CatR in *P*. *putida* is indicated with a “+” symbol. Enzymes participating in the conversion of 3-FBz to 2-FMA are identified by their corresponding EC numbers. (B) Chromosomal organization of genes encoding the *ortho*-cleavage pathway. BenABC, benzoate 1,2-dioxygenase; BenD, 1,2-dihydrodihydroxybenzoate dehydrogenase; CatA, catechol 1,2-dioxygenase; CatB, muconate cycloisomerase; CatC, muconolactone δ-isomerase; BenR, transcriptional regulator; CatR, LysR family transcriptional regulator; 1,2-DHB, 1,2-dihydrodihydroxybenzoate; 2-F-1,2-DHB, 2-F-1,2-dihydrodihydroxybenzoate; 3-F-1,2-DHB, 3-F-1,2-dihydrodihydroxybenzoate; 4-F-1,2-DHB, 4-F-1,2-dihydrodihydroxybenzoate; 5-F-DHB, 5-F-1,2-dihydrodihydroxybenzoate; 6-F-DHB, 6-F-1,2-dihydrodihydroxybenzoate; 2-FMA, 2-F-*cis*,*cis*-muconate; 3-FMA, 3-F-*cis*,*cis*-muconate; TCA cycle, tricarboxylic acid cycle. Genomic coordinates are given in bps. See also Table S1 for further details.

*P. knackmussii*^34^, ^35^, ^36^, ^37^ degrades and assimilates 2-, 3-, and 4-fluorobenzoate (2-, 3-, 4-FBz) via (1) a 1,2-dioxygenase that converts the benzoate (Bz) substrate into its 1,2-dihydroxycyclohexa-3,4-diene-1-carboxylate (1,2-dihydro-1,2-dihydroxybenzoate [1,2-DHB]) form, (2) a 1,2-DHB dehydrogenase that restores the aromatic character of the six-carbon ring structure, yielding a catechol, and (3) a second 1,2-dioxygenase that catalyzes the *ortho*-cleavage of the ring structure, resulting in (F-)*cc*MA (Figure 1A). The position of the F-substitution on the aromatic ring determines whether the corresponding compound is totally degraded (and ultimately assimilated to biomass) or not. When acting on 4-FBz, steps (1) and (2) yield 4-F-catechol (4-FC) and 3-F-*cis*,*cis*-muconate (3-FMA). This metabolite can be further processed (and defluorinated) into tricarboxylic acid (TCA) cycle intermediates. The action of benzoate-1,2-dioxygenase on 3-FBz, in contrast, can result in either 3-F-catechol (3-FC) or 4-FC—leading to 2-fluoro-*cis*,*cis-*muconate (2-FMA) and 3-FMA, respectively. Hydroxylation at the C1 and C2 ring positions of 2-FBz releases the fluoride ion (F^−^),^38^ but if the hydroxylation is performed at the C1 and C6 positions it leads to 3-FC and, consequently, to 2-FMA. This metabolite was proposed to inhibit muconate cycloisomerase, the enzyme catalyzing its further conversion, thus acting as a dead-end product.^35^ When inspecting potential biocatalysts that can produce FMAs, we noticed that the metabolic pathway from FBz to FMA in *P*. *knackmussii* conspicuously resembles the *ortho*-cleavage route in *P*. *putida*. *P*. *putida* strain mt-2 and its TOL plasmid-free variant KT2440 can degrade and consume recalcitrant xenobiotic compounds, e.g., toluene and xylenes.^39^ The *ortho*-cleavage enzymes of *P*. *putida* are encoded in two distinct gene clusters at distant locations on the chromosome. One cluster, controlled by the Bz-binding activator BenR and its associated P_*ben*_ promoter, involves the genes encoding benzoate-1,2-dioxygenase (*benA*, *benB*, and *benC*), 1,2-DHB dehydrogenase (*benD*), and several putative transporters for uptake of benzoate derivatives (*benK*, *benE-II*, and *nicP-I*),^40^ as well as catechol-1,2-dioxygenase (*catA-II*). Another catechol-1,2-dioxygenase gene (*catA*) is located within a second gene cluster, further encoding the two enzymes that process *cc*MA: muconate cycloisomerase (*catB*) and muconolactone δ-isomerase (*catC*). These genes are transcriptionally controlled by the CatR activator that responds to the intracellular *cc*MA concentration (Figure 1A).

The first enzyme complex in the cascade, formed by BenA, BenB, and BenC, is a two-component Rieske non-heme iron (Fe) dioxygenase (EC 1.14.12.10). The oxygenase component is formed by a trimer of αβ protomers (BenA and BenB) in which each α subunit contains a [2Fe-2S] cluster and a non-heme mononuclear Fe site coordinated by two histidine residues and one aspartate residue.^41^^,^^42^ BenAB has been shown to mediate high-yield, single-substrate turnover leaving both metal centers in the oxidized state. The binding of O_2_ requires both reduction of the Rieske cluster and prior substrate binding.^43^ The reductase component (BenC) is responsible for the NADH-dependent replenishing of the electrons in the benzoate-1,2-dioxygenase system with flavin adenine dinucleotide as co-enzyme and one iron-sulfur cluster of the [2Fe-2S] type.^44^^,^^45^ The reductase component of benzoate-1,2-dioxygenase of an *Acinetobacter* isolate is the only enzyme function that could be structurally resolved.^46^ Regardless, *in vitro* characterization of the substrate specificity of benzoate 1,2-dioxygenase revealed a high level of promiscuity, with significant activity on fluorinated benzoate analogs relative to the native substrate.^43^^,^^45^

The conversion of 1,2-DHB to catechol, catalyzed by the dimeric 1,2-DHB dehydrogenase BenD (EC 1.3.1.25), involves both a NAD^+^-dependent dehydrogenation and a spontaneous decarboxylation.^47^, ^48^, ^49^ Structural information on 1,2-DHB dehydrogenases is yet to be explored, and their kinetic properties have not been examined with fluorinated analogs. O_2_-dependent, oxidative cleavage of catechol is performed by two non-heme Fe(III)-containing catechol 1,2-dioxygenases (EC 1.13.11.1), CatA and CatA-II, which share 76% identity.^50^^,^^51^ CatA-II was shown to have a higher *K*_M_ for its native substrate (i.e., catechol) compared with CatA (7.4 ± 1.4 μM versus 1.3 ± 0.2 μM) as well as a lower apparent *V*_max_ (0.3 ± 0.1 μmol min^−1^ mg^−1^ versus 6.6 ± 0.3 μmol min^−1^ mg^−1^). The impact of 3- and 4-substitutions in the catechol structure on the relative activities of the respective enzymes was comparable for both CatA variants, with activities of about one-half for 4-methylcatechol, 5%–7% for 3-methylcatechol, and 2%–5% for 4-chlorocatechol.^52^ For catechol-1,2-dioxygenase from *P*. *putida* C1, the reaction rate was decreased to 21% with 4-FC compared with catechol, and the reduction in *k*_cat_ could be attributed to decreased energies in the highest occupied molecular orbital (*E*_HOMO_) in the aromatic ring in 4-substituted catechol analogs rather than the steric hindrance imposed by the substituents.^51^ Catechol 1,2-dioxygenases are evolutionarily related to the α and β subunits of *Pseudomonas* protocatechuate 3,4-dioxygenase, which has been structurally resolved.^53^

Beyond the structural and biochemical properties of these enzymes, the metabolism of FBz has been investigated in *P*. *knackmussii* by pre-incubating the cells with 3-chlorobenzoate, since fluorinated molecules cannot induce the expression of genes encoding the required catabolic activities.^35^ Two distinct catechol-1,2-dioxygenases have been identified in 3-chlorobenzoate-grown *P*. *knackmussii*.^54^ This first enzymatic step, whose regioselectivity determines which muconate derivative is formed, has a strong bias toward one of the two possible orientations depending on which halogenated substrate is fed to the cells.^55^^,^^56^ Early studies failed to identify relevant genes connected to the phenotypes observed, since the full genome of *P*. *knackmussii* was sequenced and published only by 2015.^57^ Hence, a detailed functional genomic analysis of the enzymatic complement of this bacterium was our first task toward engineering a 2-FMA-producing strain. Homology comparisons of the amino acid sequences of *P*. *putida* KT2440^58^ against the genome of *P*. *knackmussii*^57^ revealed the presence of four separated loci that harbor genes potentially relevant for FBz conversion. All genes identified in *P*. *knackmussii*, *P*. *putida* KT2440, and *P*. *putida* mt-2 (in the TOL plasmid pWW0) are listed in Table S1.

A single locus in *P*. *knackmussii* (*PKB_2100*-*PKB_2107*) encodes orthologs (>75% homology) to the *P*. *putida ben* and *cat* clusters. Two catechol-1,2-dioxygenase genes are present, one as part of the *ortho*-cleavage pathway cluster (*PKB_2107*) and the other (*PKB_1739*) in proximity to genes involved in phenol degradation (*PKB_1742*-*PKB_ 1746*). Orthologs to *catA* and *catB* (>30% homology) were found in identical copies (*PKB_3273*/*PKB_3622* and *PKB_3274*/*PKB_3623*) as part of an extended genome duplication (Figure 1B). This DNA segment, *clc*, had been previously recognized as a self-transferable element conferring the ability to degrade chloroaromatics.^59^, ^60^, ^61^ The identification and functional assignment of the relevant catabolic and regulatory genes in the haloaromatic degrader *P*. *knackmussii* set the basis for our engineering efforts in *P*. *putida*.

### *P*. *putida* outcompetes *P*. *knackmussii* as a whole-cell biocatalyst for conversion of fluoro-substituted benzoates

**Figure 2:**
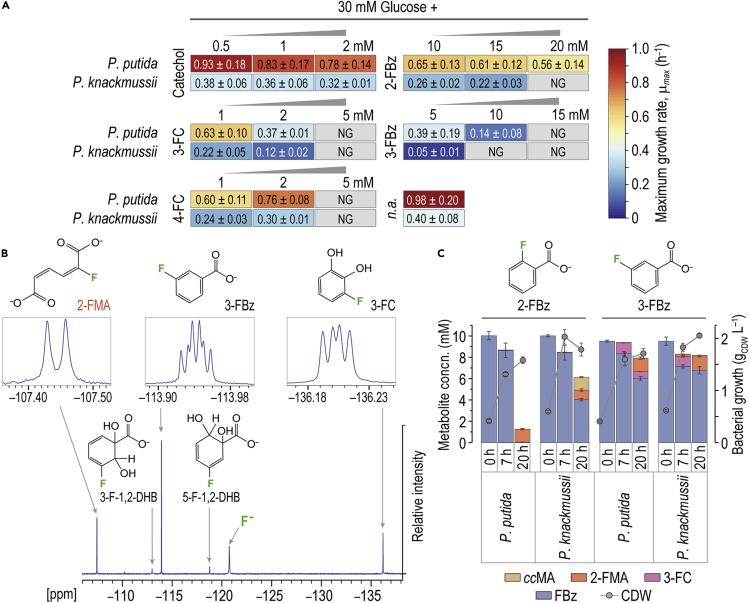
Evaluation of *Pseudomonas* species for bioconversion of fluorobenzoates (A) Growth of *P*. *putida* and *P*. *knackmussii* in the presence of fluorinated and non-fluorinated *ortho*-cleavage metabolites. Strains were cultured in microtiter plates with 30 mM glucose or benzoate and varying concentrations of the metabolites as indicated. See also Table S2 and Figure S1 for further details. (B) Coupled ^19^F-NMR spectrum of *P*. *putida* culture supernatants during 3-FBz conversion. Data represent samples taken at 20 h. (C) Utilization of fluorobenzoates by *P*. *putida* and *P*. *knackmussii*. Cells were cultured in Erlenmeyer flasks filled to 10% (v/v) with DBM medium supplemented with 30 mM glucose and 10 mM of the respective fluorobenzoate. 2-FMA, 2-fluoro-*cis*,*cis*-muconate; 3-F-1,2-DHB, 3-fluoro-1,2-dihydroxybenzoate; 3-FBz, 3-fluorobenzoate, 5-F-1,2-DHB, 5-fluoro-1,2-dihydroxybenzoate; 3-FC, 3-fluorocatechol; F^−^, free fluoride; NG, no growth; n.a., no additives; concn., concentration.

Due to the similarities observed in the enzyme repertoire of *P*. *knackmussii* and *P*. *putida*, we examined these two *Pseudomonas* species for bioconversion of 2- or 3-FBz into 2-FMA. To this end, both strains were cultured in synthetic de Bont minimal (DBM) medium with 30 mM glucose or benzoate as the main carbon source and several fluorometabolite additives (Figures 2A and S1; Table S2). We observed that 3-fluoro-*cis*,*cis-*muconate (3-FMA), produced from 4-FBz, is highly unstable due to a spontaneous cycloisomerization that releases fluoride.^62^ This occurrence renders 4-FBz as an unsuitable precursor of fluorinated *cc*MAs. Interestingly, *P*. *knackmussii* did not grow on benzoate, while *P*. *putida* grew with a maximum specific growth rate (*μ*_max_) of 0.73 ± 0.20 h^−1^. Also, *P*. *putida* grew 2.5 times faster than *P*. *knackmussii* on glucose. 2-FBz (up to 20 mM) exerted minor toxicity on *P*. *putida*, with 57% of the *μ*_max_ on glucose and without any noticeable effect on the duration of the lag phase. *P*. *knackmussii*, on the other hand, was unable to grow in the presence of 20 mM 2-FBz (at least within 50 h) and had protracted lag phases as well as reduced *μ*_max_ at 10 and 15 mM 2-FBz (Figure S1). The impact of 3-FBz on the physiology was more severe than that of 2-FBz. *P*. *putida* grew with 10 mM of this fluorometabolite, whereas *P*. *knackmussii* was inhibited at 5 mM 3-FBz and no growth was noticeable at >10 mM. Hence, growth patterns in the presence of fluorometabolites pointed to a superior performance of strain KT2440 for bioconversion. Considering the different catalytic performance of the enzymes involved on halometabolites in *P*. *putida*,^43^^,^^51^^,^^52^^,^^63^ we tested the response to catechols, potential bioconversion bottlenecks with known high toxicity.^64^ Depending on the F-substitution on benzoate, three 1,2-dihydroxybenzenes are produced as intermediary metabolites, i.e., catechol (benzene-1,2-diol), 3-fluorocatechol (3-fluorobenzene-1,2-diol; 3-FC), and 4-fluorocatechol (4-fluorobenzene-1,2-diol; 4-FC). No significant toxicity was observed when *P*. *putida* or *P*. *knackmussii* were incubated in the presence of 2 mM catechol. The physiological response to the two FC derivatives was much more pronounced, with 3-FC substantially reducing *μ*_max_ (Figure S1), but *P*. *putida* exhibited higher resistance to both 3-FC and 4-FC than *P*. *knackmussii*.

Next, the two species were subjected to a fermentation experiment with 2-FBz or 3-FBz, whereby all fluorometabolites are endogenously produced from these substrates (rather than externally added). Supernatants were analyzed via high-resolution ^19^F nuclear magnetic resonance (^19^F-NMR) spectroscopy (Figure 2B). Signals at −107.5, −113.0, −113.9, −118.9, and −136.2 ppm correspond to 2-FMA, 3-F-DHB, 3-FBz, 5-F-DHB, and 3-FC, respectively. The free F^−^ signal was observed at ca. −121 ppm. The chemical identity of all fluorometabolites was confirmed by ^1^H,^1^H-^13^C heteronuclear single quantum coherence spectroscopy (1-bond ^1^H-^13^C correlations) and ^1^H-^13^C heteronuclear multiple bond correlation (2- to 4-bond ^1^H-^13^C correlations) NMR experiments. Furthermore, 2-FBz, 3-FBz, catechol, 3-FC, 4-FC, and *cc*MA were quantified via high-performance liquid chromatography (HPLC) against commercial standards (Figure S2). *P*. *putida* completely consumed 2-FBz within 20 h (Figure 2C), with 2-FMA as the only fluorometabolite detectable upon substrate depletion. *P*. *knackmussii* only processed 60% of the haloaromatic, and the conversion stalled after 20 h. 2-FMA was produced to just 1 mM together with *cc*MA, which continued to be consumed until the end of the experiment. For both species, 2-FMA amounted to ca. 14% of the 2-FBz consumed (i.e., molar product yield *Y*_P/S_ = 0.14 mol mol^−1^). 3-FBz, on the other hand, was only partially consumed by *P*. *putida*. All 3-FBz was converted into 3-FC within the first 7 h, leading to a pinkish coloration of the medium. Accumulation of FCs ≥0.2 mM caused spontaneous auto-oxidation followed by formation of colored polymerization products, similar to non-fluorinated catechols.^65^ By the end of the experiment, 3-FBz had been converted into 2-FMA with *Y*_P/S_ = 0.41 mol mol^−1^, as well as a residual amount of 3-FC. Both 2-FMA and 3-FC represented 55% (mol mol^−1^) of the 3-FBz consumed—the remainder was defluorinated and assimilated into biomass (Figure 1A). *P*. *knackmussii* partially converted 3-FBz (without prior incubation with chlorobenzoate) concomitant with an initial increase of 3-FC, which was reconsumed until 20 h and transformed into 2-FMA, ultimately leading to *Y*_P/S_ = 0.55 mol mol^−1^. Unbalanced catalytic rates led to a two-stage conversion profile for both species, with the 3-FBz → 3-FC and 3-FC → 2-FMA phases temporally decoupled. In addition, no 4-FC could be detected in the culture supernatants in any condition. Thus, the enzymes performing *ortho*-cleavage in both pseudomonads seem to have a higher activity on 4-FC than 3-FC, explaining the lesser growth-inhibiting effect of 4-FC.

Taken together, the results in this section indicate that (1) the non-productive 1,2-dioxygenation on 2-FBz causes an 85% loss of F as F^−^, hence 3-FBz is the most suitable precursor to 2-FMA, and (2) *P*. *putida* stands out as a whole-cell biocatalyst for FBz bioconversion into 2-FMA, with faster *μ*_max_ and higher resistance to toxic pathway intermediates. 3-FC toxicity, however, constitutes a significant bottleneck for an efficient, prolonged bioprocess using *P*. *putida*. Based on these observations, and the transcriptional architecture of the gene clusters associated with the relevant biochemical activities, a pathway-balancing approach was pursued to relieve metabolic bottlenecks as explained in the next section.

### Engineering *P*. *putida* to increase 3-fluorobenzoate bioconversion

**Figure 3:**
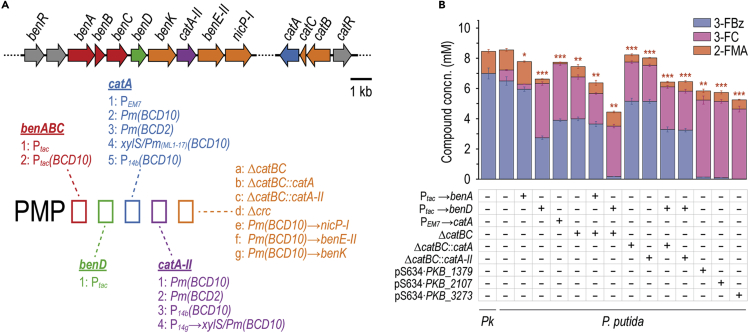
Classification and performance of first-generation engineered PMP strains in 3-FBz bioconversion (A) Nomenclature of engineered 2-FMA-producing *P. putida* (PMP) strains, represented along the gene clusters in strain KT2440 that encode relevant enzymatic activities. (B) Bioconversion performance of first-generation engineered strains in shaken-flask fermentations. The strains were cultured in Erlenmeyer flasks filled to 10% (v/v) with DBM medium with 30 mM glucose and 10 mM 3-FBz. The concentration of extracellular metabolites was measured after 24 h. Statistically significant changes in 2-FMA concentrations of the strains compared with wild-type *P*. *putida* were determined by Student's *t* test (two-sample, unpaired), with ∗p < 0.05, ∗∗p < 0.01, ∗∗∗p < 0.001. *Pk*, *Pseudomonas knackmussii*; concn., concentration.

Synthetic constitutive promoters were implemented to drive expression of key genes in an attempt to increase the availability of bioconversion enzymes (especially catechol 1,2-dioxygenases). The nomenclature adopted for engineered 2-FMA-producing *P*. *putida* (PMP) strains is illustrated in Figure 3A, and all metabolic modifications are listed in Table S3—i.e., replacing the native P_*ben*_ promoter upstream *benA* with P_*tac*_, inserting P_*tac*_ upstream of *benD*, and adding the P_*EM7*_ promoter upstream of *catA*. Inserting regulatory elements comprising P_*tac*_ and a translational coupler (bicistronic designs *BCD2* or *BCD10*)^66^ upstream of *catA* or *catA-II* consistently led to mutations within the −35 region of P_*tac*_. This suggests a toxic effect of constitutive overexpression of catechol 1,2-dioxygenase genes. As an alternative strategy to increase this catabolic activity, the three *catA* homologs from *P*. *knackmussii* were cloned into vector pSEVA634, controlled by an isopropyl-β-D-1-thiogalactopyranoside-inducible LacI^*q*^/P_*trc*_ element, and inserted into *P*. *putida*. Further strains contained in-frame *catB* and *catC* deletions to avoid product degradation. In additional strain variants, *catBC* were replaced by *catA* or *catA-II*, placing them under control of the native CatR/P_*cat*_ system.

Each of the first-generation PMP strains was incubated in DBM medium with 30 mM glucose and 10 mM 3-FBz. All cultures reached a terminal state within 24 h, whereby no further 3-FBz was consumed and all fluorometabolites remained constant. Comparing these states allowed us to analyze the (im)balance of biochemical activities involved in 2-FMA biosynthesis (Figure 3B). As hinted at already, the pathway in *P*. *putida* is clearly not optimized to process haloaromatics, as made apparent by significant accumulation of 3-FC in all experiments thus far. This fluorometabolite was only slightly reduced in the strain where the P_*tac*_ promoter replaced P_*ben*_. Insertion of P_*tac*_ upstream of *benD* increased 3-FBz consumption but led to a 5-fold higher 3-FC formation than in wild-type *P*. *putida*—amplifying, rather than solving, the metabolic bottleneck around this fluorometabolite. Importantly, the high flux toward 3-FC points to a rate-limiting role of either BenD or the BenK benzoate/H^+^ symporter. Overexpressing *catA*^52^ by means of P_*EM7*_ increased substrate consumption in strain PMP0100 but resulted in a nearly stoichiometric conversion of 3-FBz to 3-FC. The deletion of catBC also caused high accumulation of 3-FC in the respective strains. Implementing additional *catA* or *catA-II* copies under P_*cat*_ in place of *catBC* did not show any positive effect on the conversion of 3-FC into 2-FMA. Expression of *P*. *knackmussii catA* orthologs from medium-copy-number plasmid pSEVA634 greatly enhanced 3-FBz uptake, with nearly all substrate consumed within 24 h. This effect, again, caused 50% of the substrate to be converted to 3-FC. Hence, it became clear that FC detoxification remained a bottleneck for the catalytic performance in all these PMP strains and, out of the manipulations tested, only P_*tac*_ → *benABC* had a mild pathway-balancing effect. Synthetic transcriptional control of *benABC* or *benD* brought about comparable effects in a *ΔcatBC* background as it did in *catBC*^+^ strains. Furthermore, efforts to increase the enzyme abundance of catechol 1,2-dioxygenase appeared to reduce the rate at which the biochemical reaction was performed while enhancing 3-FBz consumption. These results indicate that a more finely orchestrated transcriptional control of the relevant genes is needed to boost catalytic performance.

### Characterization of expression devices to balance 3-fluorobenzoate bioconversion

**Figure 4:**
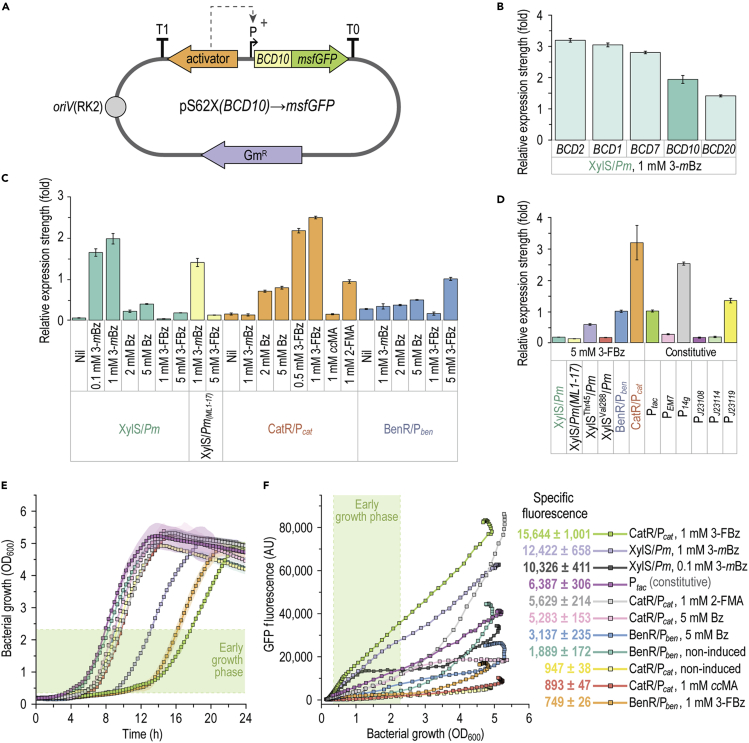
Transcriptional activity of native *P*. *putida* expression systems in response to fluorometabolites Inducible systems (XylS/*Pm*, BenR/P_*ben*_, and CatR/P_*cat*_) and constitutive promoters (P_*tac*_, P_*EM7*_, P_*14g*_, P_*J23108*_, P_*J23114*_, and P_*J23119*_), alone or in combination with *BCD* sequences, were cloned upstream of *msfGFP* in vector pSEVA627M and delivered into *P*. *putida*. Strains were cultured in DBM medium with 30 mM glucose and varying concentrations of fluorometabolites. Expression strength was normalized to that of the P_*tac*_ promoter; in all cases error bars represent standard deviations of average values from three biological replicates. Nil, no co-inducer compound added. (A) General structure of reporter plasmids constructed to characterize the expression systems. Various promoters (P), the translational coupler *BCD10*, and, if applicable, cognate regulators, were cloned upstream the *msfGFP* reporter gene in vector pSEVA627M, where the cargo segment is flanked by two terminators (T_0_ and T_1_). Gm^R^, gentamicin-resistance determinant. (B) Efficiency of *BCD* sequences in initiating mRNA translation. (C) Inducibility of promoter systems controlling *ortho*-cleavage pathway genes and the XylS/*Pm* system. Each promoter sequence was added with a *BCD10* element to initiate translation. (D) Relative transcriptional strengths of promoter systems used for strain engineering under bioconversion conditions with *BCD10* as translation initiation sequence. (E) Growth of *P*. *putida* KT2440 with selected reporter plasmids and conditions, estimated as the optical density measured at 600 nm (OD_600_). (F) msfGFP fluorescence for selected strains and conditions. Slopes (i.e., specific fluorescence) were determined via linear regression during exponential growth (boxed in light green; the slope for XylS/*Pm* with 0.1 mM 3-*m*Bz was determined up to an OD_600_ of 1.2). AU, arbitrary units.

To understand the effects of transcriptional manipulations in first-generation PMP strains and to guide the next set of modifications, we subjected all regulatory DNA sequences relevant for the bioconversion process to a quantitative characterization (Figure 4). To this end, a set of reporter plasmids was constructed to explore the activity of different catabolic promoters that could play a role in FBz utilization. Vector pSEVA627M was adopted as the backbone for all constructs.^67^ Besides the low-copy-number origin of replication *oriV*(RK2) and a gentamicin-resistance determinant, each plasmid harbored the *msfGFP* reporter gene under control of the bicistronic translational coupling sequence *BCD10* (Figure 4A).^66^ Transcriptional control was exerted by the *Pm*, P_*ben*_, or P_*cat*_ promoters together with their respective cognate activator proteins XylS, BenR, or CatR. Further included in the analysis was a set of constitutive promoters with different strengths and variants of the XylS/*Pm* system (Table S4). Wild-type *P*. *putida*^67^, ^68^, ^69^, ^70^, ^71^ harboring each of the reporter plasmids was cultured in DBM medium with 30 mM glucose and, if applicable, co-inducer (fluoro)metabolites. First, a comparison of effects brought about by different *BCD* sequences is shown in Figure 4B. The *BCD* variants initiated translation with relative rates as follows: *BCD2* > *BCD1 > BCD7 > BCD10 > BCD20*. Hence, with this set of sequences, the expression of target genes can be tuned within a ca. 2.5-fold range. Figure 4C compares the transcription strength of the native XylS/*Pm*, CatR/P_*cat*_, and BenR/P_*ben*_ systems, as well as *Pm*_(*ML1-17*)_, a promoter variant reported to be stronger than wild-type *Pm*.^72^ The induction strength afforded by 3-*m*Bz, the native *ortho*-cleavage substrate Bz, the bioconversion substrate 3-FBz, and, for CatR/P_*cat*_, *cc*MA and 2-FMA, was systematically assessed with this toolset.

With both 3-*m*Bz and 3-FBz, *msfGFP* expression under *Pm*_(*ML1-17*)_ amounted to <80% of the level provided by wild-type *Pm*. XylS/*Pm* was almost fully induced by 0.1 mM 3-*m*Bz during the first 3 h of cultivation, with the maximum optical density measured at 600 nm (OD_600_)-normalized fluorescence values reaching about half of that observed with 1 mM 3-*m*Bz. In contrast, Bz and 3-FBz set a linear dependency between inducer concentration and the msfGFP signal in the 1- to 5-mM range, with a fluorescence output <20% of that elicited by 1 mM 3-*m*Bz. The XylS/*Pm* system did not react to 1 mM 3-FBz. CatR/P_*cat*_ had the highest expression strength and was almost entirely induced with 0.5 mM 3-FBz while 3-*m*Bz elicited no response. Only one-third of fluorescence was observed with 5 mM Bz compared with 3-FBz. Furthermore, the system responded 7-fold more strongly to 1 mM 2-FMA than to 1 mM *cc*MA. CatR/P_*cat*_ was 2.7-fold stronger with 3-FBz than with 2-FMA, indicative of more efficient uptake of 3-FBz followed by intracellular conversion to 2-FMA. The more vigorous response to 3-FBz and 2-FMA compared with Bz and *cc*MA could be caused either by a stronger CatR interaction with the fluoro-substituted muconate or by a more substantial 2-FMA accumulation, as *cc*MA is further assimilated into biomass.

The BenR/P_*ben*_ system displayed high basal expression, and only a slight increase in msfGFP could be observed with 1 mM 3-*m*Bz and 2 mM Bz. With 5 mM Bz, the output was increased by 81% compared with the non-induced system. 3-FBz caused no effect on BenR/P_*ben*_ at 1 mM. However, at 5 mM, 3-FBz triggered an msfGFP production 2-fold higher than with 5 mM Bz. In this sense, overproduction of BenR is known to cause activation from its associated promoter in the absence of inducers through spontaneous dimerization.^73^^,^^74^ Hence, increasing *benR* copies via plasmid expression could be responsible for the high basal output observed herein. In addition, the translation of BenR mRNA in *P*. *putida* is inhibited by Crc, the catabolite repression control protein. This notion is supported by the course of *msfGFP* output over the growth curves (Figures 4E and 4F). While there was a linear increase of fluorescence in the early exponential phase, it rose exponentially within the mid-to-late growth phase and continued into the early stationary phase. Once glucose is depleted, Crc no longer represses genes involved in assimilation of alternative carbon sources.^75^ An alternative explanation for the differential behavior of BenR/P_*ben*_ is the recruitment of different σ factors by P_*ben*_ at different growth stages, as observed with the highly homologous *Pm* promoter. *Pm* recruits σ^32^- and σ^38^-dependent RNA polymerases,^76^ with σ^32^ being active predominantly within exponential growth and σ^38^ activating transcription in stationary phase.^77^

The inducible systems were compared with the constitutive P_*tac*_, P_*EM7*_, P_*14g*_, P_*J23108*_, P_*J23114*_, and P_*J23119*_ promoters under 2-FMA production conditions with 5 mM 3-FBz (Figure 4D). The XylS^Thr45^ and XylS^Val288^ variants had been described to respond more strongly to 3-substituted Bz derivatives,^78^ and were included in the analysis to explore 3-FBz as an effector. In our experiments, the transcription initiation rates with 5 mM 3-FBz relative to P_*tac*_ ranked as follows: CatR/P_*cat*_ (3.2) > P_*14g*_ (2.5) > P_*J23119*_ (1.3) > P_*tac*_ (1.0) = BenR/P_*ben*_ (1.0) > XylS^Thr45^/*Pm* (0.6) > P_*EM7*_ (0.3) > P_*J23114*_ (0.2) = P_*J23108*_ (0.2) = XylS/*Pm* (0.2) = XylS^Val288^/*Pm* (0.2) > XylS/*Pm*_(*ML1-17*)_ (0.1). The expression strengths of the systems employed in the first round of strain engineering explain the phenotypes observed. At high 3-FBz concentrations, BenR/P_*ben*_ provided an expression level comparable with that of P_*tac*_. The slightly increased consumption observed in strain PMP1000 compared with wild-type strain KT2440 (Figure 3B) may be attributed to full activity of the *ben* operon supported by P_*tac*_ at the onset of the cultivation, whereas BenR/P_*ben*_ is induced only upon substrate exposure. In contrast, CatR/*P*_*cat*_, which controls *catA* transcription, was the strongest system responding to 3-FBz. Relevant to this observation, *catA-II* is likely expressed together with *benK* as a polycistron (Figure S3). This operon is presumably subject to Crc regulation, indicated by recognition motifs for Hfq,^79^ which promotes Crc binding to its targets.^80^ Any effort to increase catechol 1,2-dioxygenase gene expression resulted in the detrimental accumulation of 3-FC, suggesting a decrease in the respective catalytic activity as a consequence of our manipulations. Taken together, the induction pattern of the expression systems and the bioconversion performance of first-generation strains suggest that a reduction—rather than an increase—in *catA*(*-II*) expression may increase bioconversion efficiency in *P*. *putida*.

### Dynamic control of catechol 1,2-dioxygenase enables complete conversion of 3-fluorobenzoate to 2-fluoro-*cis*,*cis*-muconate

A second generation of PMP strains was designed (Table S3), guided by physiological observations with first-generation strains and the characterization of expression systems described above. Here, *catA* or *catA-II* was placed under XylS/*Pm* control, which responded weakly to 3-FBz (thereby providing a more balanced output) with almost zero transcriptional leakiness.^73^ Because 3-FBz-induced XylS/*Pm* affords substantially lower transcript levels compared with native CatR/P_*cat*_ (Figure 4D) and both 5′ untranslated regions (UTRs) of *catA* and *catA-II* are subject to catabolite repression, the endogenous *TIS*s of both genes were replaced by *BCD10* or *BCD2* to increase translation rates. These changes were combined either with P_*tac*_ *→ benABC* or chromosomal *xylS* integration under control of its own regulatory signals. Additionally, some strains were transformed with vector pSEVA228 to provide the XylS activator in *trans*.

**Figure 5:**
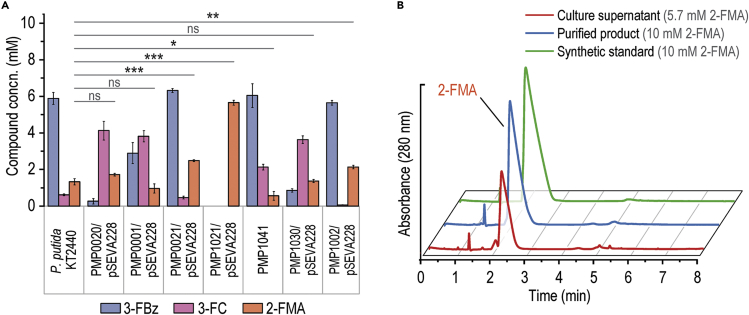
Performance of second-generation engineered PMP strains in 3-FBz bioconversion Strains were cultured in Erlenmeyer flasks filled to 10% (v/v) with DBM medium and supplemented with 30 mM glucose and 10 mM 3-FBz. (A) Pattern of extracellular fluorometabolites after 24 h of cultivation. Error bars represent standard deviations of average values from three biological replicates. Statistically significant changes in 2-FMA concentrations of engineered strains compared with *P*. *putida* KT2440 were determined by Student's *t* test (two-sample, unpaired), with ∗p < 0.05, ∗∗p < 0.01, ∗∗∗p < 0.001. ns, not significant; concn., concentration. (B) HPLC chromatograms (UV detection at 280 nm) of a 24-h culture medium of strain PMP1021/pSEVA228, 2-FMA purified from supernatants, and a chemically synthesized 2-FMA standard.

Second-generation strains were screened as described above, and the exometabolome was quantified in 24-h cultures (Figure 5A). While individually replacing the native *catA* and *catA-II* regulatory sequences with *Pm*(*BCD10*) (in strains PMP0020/pSEVA228 and PMP0001/pSEVA228) led to increased 3-FC formation, both manipulations combined (strain PMP0021/pSEVA228) reduced the levels of this bottleneck fluorometabolite and boosted 2-FMA biosynthesis by 2-fold compared with *P*. *putida* KT2440. Yet ca. 50% of 3-FBz was left untouched in the culture medium. This occurrence was remediated by the additional implementation of P_*tac*_ *→ benABC* in strain PMP1021/pSEVA228, which enhanced 3-FBz uptake and led to complete substrate transformation with *Y*_P/S_ = 0.5 mol mol^−1^ and without 3-FC accumulation—i.e., the maximum theoretical yield. 2-FMA was the only fluorometabolite detected in cultures of strain PMP1021/pSEVA228 (Figure 5B), and the broth was subjected to purification by adapting a protocol established for *cc*MA.^15^ The procedure entails microfiltration, treatment with activated charcoal, crystallization by pH/temperature shifts, vacuum filtration, and vacuum drying. When this sequence was applied to our samples, it yielded ca. 35 mg of 2-FMA from 45 mL of culture broth (84% recovery). HPLC analysis of the purified, isomerically pure product coupled to UV detection matched that of a chemically synthesized mixture of (2*Z*,4*Z*), (2*Z*,4*E*), (2*E*,4*Z*), and (2*E*,4*E*) 2-FMA isomers (Figure 5B), with a single absorbance maximum at 269 nm^35^ and structural identity confirmed via ^19^F-NMR.

**Figure 6:**
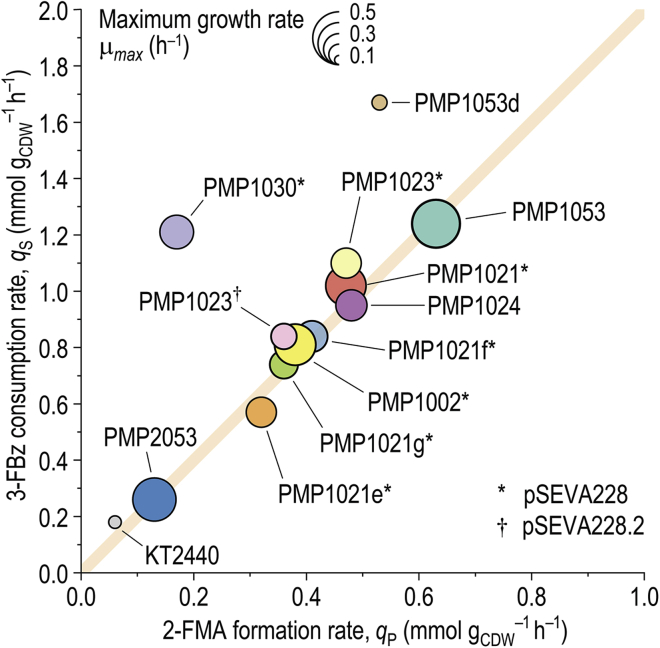
Performance parameters for selected strains used in this study in the bioconversion of 10 mM 3-FBz To determine the biomass-specific 3-FBz uptake rates (*q*_S_) and 2-FMA formation rates (*q*_P_), the strains were cultured in Erlenmeyer flasks filled to 10% (v/v) with DBM medium supplemented with 30 mM glucose and 10 mM 3-FBz. Maximum growth rates (*μ*_max_), indicated by the relative size of the circles, were determined in microtiter plate experiments in the same culture medium and growth conditions. A *q*_P value_ of ½ × *q*_S_ indicates a balanced bioconversion at the maximum theoretical yield (as indicated by an orange diagonal). See also Table S5 for specific values.

The enhanced catalytic performance of second-generation PMP strains could only be observed if XylS was provided from plasmid pSEVA228, as revealed by the accumulation of 3-FC by strain PMP1041. Thus, relatively high levels of the regulator are necessary to saturate both *Pm* promoters of *catA* and *catA-II*, located at distant chromosome loci. This observation is in line with the pattern of XylS transcriptional output, known to be more heterogeneous within a cell population at increased regulator gene-*Pm* promoter distances.^73^^,^^81^ We further attempted to dissect which *catA* paralog has the most impact on the bioconversion. While expression of *catA* via *Pm*(*BCD2*) (strain PMP1030/pSEVA228) led to 3-FC accumulation, tuning the expression of *catA-II* (strain PMP1002/pSEVA228) enabled stoichiometric conversion of 3-FBz into 2-FMA, albeit at a lower productivity than with strain PMP1021/pSEVA228 (Figure 6). This result suggests that CatA-II is the dioxygenase predominantly responsible for converting 3-FC into 2-FMA. Seeking to enhance 3-FBz uptake, each of the three benzoate transport-associated genes (i.e., *benK*, *benE-II*, or *nicP-I*) was placed under *Pm*(*BCD10*) control in a PMP1021/pSEVA228 strain background. While balanced conversion was maintained in each of these strains, *q*_S_ was significantly reduced (Figure S4). The evidence gathered thus far guided the design of the third generation of PMP strains to enhance both kinetic parameters while avoiding the use of plasmids.

### Constitutive expression of catechol 1,2-dioxygenase genes balances 2-fluoro-*cis*,*cis*-muconate biosynthesis in plasmid-free engineered strains

**Figure 7:**
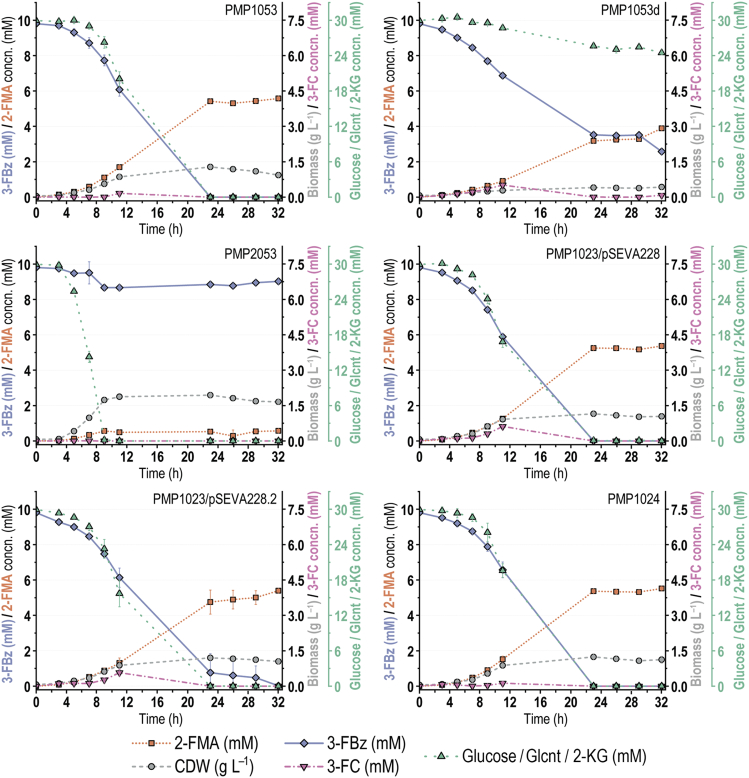
Performance of third-generation engineered PMP strains in bioconversion of 3-FBz to 2-FMA Experiments were performed in Erlenmeyer flasks filled to 10% (v/v) with DBM medium supplemented with 30 mM glucose and 10 mM 3-FBz. Error bars represent standard deviations from three biological replicates around arithmetic averages. Glcnt, gluconate; 2-KG, 2-ketogluconate; CDW, cell dry weight; concn., concentration.

Another set of PMP strains was designed to characterize the effects of P_*tac*_(*BCD10*) *→ benABC* and Δ*crc* to enable 3-FBz bioconversion without the use of plasmids and to consolidate the contributions of *catA* and *catA-II* to 3-FC detoxification (Table S3). In particular, in strains PMP1053, PMP1053d, PMP2053, and PMP1023/pSEVA228(.2), either *catA-II* or both *catA* genes were placed under the constitutive P_*14b*_ promoter (which has 25% of the strength of P_*14g*_)^82^ and a *BCD10* element. Also, *xylS* was integrated under the P_*14g*_ promoter together with a synthetic *TIS* designed with the RBS Calculator (engineered in strain PMP1024). Fermentation profiles of third-generation PMP strains, grown as indicated previously, are shown in Figure 7. The combination of P_*tac*_ → *benABC*, P_*14b*_(*BCD10*) → *catA*, and P_*14b*_(*BCD10*) → *catA-II* in strain PMP1053 enabled complete conversion of 3-FBz (*q*_S_ = 1.24 ± 0.01 mmol g_CDW_^−1^ h^−1^) at maximum theoretical yield and highest specific productivity (*q*_P_ = 0.63 ± 0.04 mmol g_CDW_^−1^ h^−1^) in the absence of any plasmid (Figure 6; Table S5). Furthermore, the constitutive expression of *catA* and *catA-II* decreased the initial 3-FC accumulation compared with that in strain PMP1023/pSEVA228, in which catechol 1,2-dioxygenases were activated only upon exposure to 3-FBz. Notably, with both *catA* and *catA-II* constitutively expressed, engineering P_*tac*_(*BCD10*) control for *benABC* (strain PMP2053) resulted in an incomplete consumption of 3-FBz at low *q*_S_. In contrast, deletion of *crc* (strain PMP1053d) yielded the highest 3-FBz-specific *q*_S_ observed in this study. Simultaneously, the strain demonstrated a striking decrease in glucose consumption, limiting the supply of catalytic biomass. In line with these observations, *catA* transcriptional levels should be kept within a narrow range toward efficient bioconversion. In strain PMP1023, for instance, only *catA-II* was constitutively expressed, while *catA* was under *Pm*(*BCD10*) regulation. For exploration of different *catA* induction strengths, XylS was provided either in its wild-type form (plasmid pSEVA228) or as the XylS^Thr45^ variant (plasmid pSEVA228.2), which caused a 3-fold higher induction of *msfGFP* in response to 3-FBz than XylS (Figure 4). However, strain PMP1023 had a ca. 20% higher *q*_P_ with plasmid pSEVA228 than with pSEVA228.2 (Figure 6; Table S5). Thus, vector pSEVA228 was kept in plasmid-containing strain design.

Prompted by these results, chromosome-based expression of the *xylS* regulator gene was tested in another series of engineered strains. Unlike strain PMP1041, in which *xylS* was integrated upstream of *catA*, landing the regulator gene in proximity to *catA-II* (strain PMP1024) provided sufficient expression from the chromosomal *Pm* promoters to enable a complete 3-FBz bioconversion. The *q*_S_ and *q*_P_ values in this case were comparable with those of strain PMP1021/pSEVA228 (Table S5), without the necessity for a plasmid. Hence, the results with PMP1023 and PMP1024 confirm the previous notion that the control of *catA-II* expression is the predominant mechanism that allows for efficient 3-FC detoxification. The combinatorial pathway balancing thus solved the metabolic bottleneck around this fluorometabolite, suppressing the temporally decoupled profile of 3-FBz bioconversion. Considering these results, strain PMP1053 was kept for further experiments as the best-performing biocatalyst for 2-FMA biosynthesis.

### Fully engineered *P*. *putida* tolerates high bioconversion substrate levels

**Figure 8:**
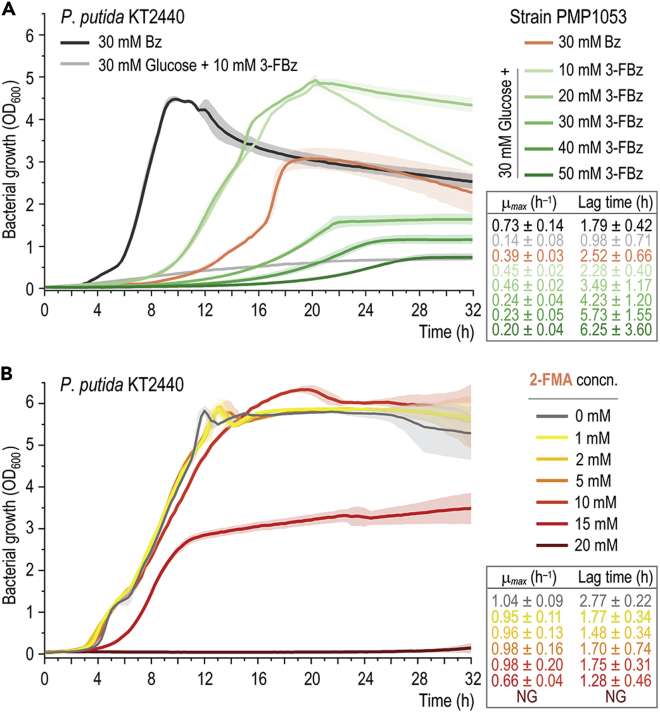
Toxicity of the bioconversion substrate and product for wild-type and engineered *P*. *putida* *P*. *putida* KT2440 and engineered strain PMP1053 were cultured in microtiter plates in 200 μL of DBM medium and various additives as indicated. (A) Growth patterns and kinetic parameters of strains KT2440 and PMP1053 on 30 mM benzoate (Bz) or 30 mM glucose in the presence of increasing 3-FBz concentrations. (B) Growth patterns and kinetic parameters of strain KT2440 with 30 mM glucose and increasing 2-FMA concentrations. Error bars represent standard deviations from three biological replicates. NG, no growth; concn., concentration.

We reasoned that the superior kinetic performance of strain PMP1053 could be harnessed to push the limits of substrate that can be used in the biotransformation process, previously identified as a limiting step toward high 2-FMA output (Figure 3). Therefore, strain PMP1053 was first tested regarding its ability to use Bz as the sole carbon source and the maximum 3-FBz concentrations tolerated in the presence of 30 mM glucose as the primary substrate (Figure 8A). 3-FBz exerted no toxic effects at concentrations up to 20 mM, while the growth of wild-type *P*. *putida* KT2440 was entirely suppressed by 10 mM 3-FBz. Moreover, strain PMP1053 grew in the presence of up to 50 mM 3-FBz, although at a reduced *μ*_max_ and maximum biomass concentration. Besides, the higher the initial 3-FBz concentration, the longer the lag phase (up to ca. 6 h in the presence of 50 mM of the bioconversion substrate). The apparent increase in OD_600_ in cultures of wild-type KT2440 could be attributed to the formation of colored FC polymerization products. Importantly, no medium coloration was observed in cultures of strain PMP1053, indicating that the 3-FBz consumed was assimilated without any transient 3-FC formation. The apparent lack of 3-FC accumulation and the fact that growth deficiencies (reflected in *μ*_max_) were noticed at the onset of the cultivation suggests a toxic effect on the cells by 3-FBz itself. Interestingly, the *μ*_max_ and biomass yield of strain PMP1053 grown on benzoate were significantly reduced compared with wild-type *P*. *putida* KT2440 (by 50% and 30%, respectively). These observations highlight that the combinatorial pathway-balancing strategy is tailored for 3-FBz processing (as opposed to any benzoate substrate).

Since the wild-type strain and the first and second generations of PMP variants had a very low 2-FMA output, assessing toxicity of the endogenously produced product was obviously not possible. Building on the results obtained with the best-performing engineered strains, the toxicity of 2-FMA was directly investigated in DBM medium with 30 mM glucose as the main carbon source and varying concentrations of the bioconversion product (Figure 8B). *P*. *putida* KT2440 showed a growth profile similar to that of cultures with glucose as the only additive in the medium with up to 10 mM 2-FMA, indicating that the bioconversion product is less toxic than the bottleneck fluorometabolite 3-FC. At 15 mM 2-FMA, the *μ*_max_ and the maximum biomass concentration were approximately halved, and no growth was detected at 20 mM 2-FMA or above. Interestingly, when exposed to 2-FMA, strain PMP1053 had a growth profile nearly identical to that of the wild-type strain (not shown). Because 2-FMA exerts a stronger toxic effect on engineered *P*. *putida* than 3-FBz, upscaling of the bioconversion process will likely be limited by the concentrations of the product. However, 2-FMA tolerance can likely be readily improved by methods of adaptive laboratory evolution.^83^, ^84^, ^85^ Taken together, these results indicate that not only did strain PMP1053 have the best performance among all engineered *P*. *putida* variants in terms of kinetic parameters, but it also had superior tolerance to toxic fluorometabolites (especially the bioconversion substrate). These properties render this fully engineered strain suitable for exploring the biosynthesis of new-to-industry fluorinated building blocks, harnessing the rich metabolic capacity and stress tolerance of this species.

## Discussion

### The divergent metabolic specialization of *P*. *putida* and *P*. *knackmussii* results in distinct processing patterns for benzoate and its fluorinated derivatives

Both wild-type *P*. *putida* and *P*. *knackmussii* accumulated 3-FC when exposed to 3-FBz, but 4-FC could not be detected in these cultures, whereas the relative production rates of both intermediates were comparable (due to the regioselectivity of BenABC). Thus, the catechol 1,2-dioxygenases from both species display higher activities on the 4-substituted catechol than on the 3-substituted derivative. 3-FC, produced in the early phase of incubation, was fully converted into 2-FMA by *P*. *knackmussii*, whereas *P*. *putida* failed to detoxify this bottleneck fluorometabolite. Besides, *P*. *knackmussii* could not grow on benzoate as sole carbon source (in contrast to *P*. *putida*, which grew well on this substrate). Conversely, when *P*. *putida* was engineered to process haloaromatics, benzoate-dependent growth was impaired, indicating that combinatorial pathway-balancing approaches are substrate specific and should be adjusted depending on the target product.

From a more general perspective, our results expose the different evolutionary specialization of the two pseudomonads in utilizing non-halogenated and halogenated *ortho*-cleavage substrates and indicate contrasting regulatory and biochemical requirements for the two types of substrate. This aspect is further illustrated by the presence of a second catechol 1,2-dioxygenase in strain KT2440, proposed to serve as a safety valve to deal with toxic catechols.^52^ CatA-II has a lower affinity and activity on its native substrate than CatA, while two paralogs' catalytic activities were similarly affected by methyl- and chloro-substituted arenes. In our experiments, fine-tuning *catA-II* expression alone afforded sufficient balancing of the overall flux through the pathway to enable complete conversion of 3-FBz to 2-FMA. These results suggest an essential role of *catA-II* in the conversion of 3-fluorocatechol, a substrate, which so far has not been included in studies characterizing catechol 1,2-dioxygenases. *P*. *knackmussii* also harbors two putative catechol 1,2-dioxygenases with significantly higher homology to the *P*. *putida* CatA proteins as well as close homologs to the remaining *ortho*-cleavage enzymes, and growth on benzoate was reported in the literature. Therefore, it seems plausible that regulation of the gene functions rather than the enzymatic capabilities are responsible for the observed lower productivities on (F)Bz and that an engineering approach similar to that used in *P*. *putida* could lead to efficient *P*. *knackmussii* cell factories.

### Overexpression of catechol 1,2-dioxygenase genes simultaneously enhances 3-fluorobenzoate consumption and decreases 3-fluorocatechol detoxification

Debottlenecking 3-FC was initially addressed via constitutive *catA* overexpression, which greatly enhanced 3-FBz consumption. However, a mere “pulling” effect (i.e., removal of toxic pathway intermediates) seems unlikely, since we also detected decreased conversion of 3-FC to 2-FMA. van Duuren et al.^86^ demonstrated a suppressive effect of catechol on transcription from the P_*ben*_ promoter in a CatR-deficient *P*. *putida* strain. 3-FC exerted an altogether different effect. Although wild-type *P*. *putida* and engineered PMP1053 accumulated significantly different amounts of this intermediate when exposed to 3-FBz, *msfGFP* expression was comparable in both strains harboring a BenR/P_*ben*_ → *msfGFP* construct (data not shown). Since F engages in chemical interactions different from those in hydrogen, fluorinated ligands expectedly display altered affinity to their receptors compared with non-halogenated counterparts.^87^, ^88^, ^89^ In some cases, the presence of even a single F atom in a substrate surrogate results in a strong inhibitory effect on enzyme activity.^90^ The *ortho*-cleavage pathway has been traditionally studied with its native substrates,^91^ which explains why halogenated effectors mediate transcriptional and metabolic responses different from those observed with natural substrates and intermediates.

### Targeted replacement of regulatory sequences governing expression of key *ortho*-cleavage pathway genes reveals hidden control layers

The expression of *ben* genes changed drastically depending on the cell metabolic state, as seen in glucose-grown *P*. *putida* expressing *msfGFP* under BenR/P_*ben*_ control. A similar effect of physiological control mechanisms was reported for CatR/P_*cat*_.^92^ Reporter activity of P_*ben*_ and P_*cat*_ fusions or the abundance of *benA* and *catB* transcripts were commonly used as a proxy of the expression of the whole gene clusters.^92^, ^93^, ^94^, ^95^, ^96^ The picture on regulatory mechanisms controlling the expression of all eight genes in the *ben* cluster or the three genes in the *cat* cluster is far from complete. Transcriptomic data published for *P*. *putida* grown on glucose, fructose, glycerol, and succinate or exposed to toluene or ferulate revealed potential, hitherto unknown transcription start sites within the *ben* cluster.^97^ Furthermore, Hfq binding motifs are found upstream of several *ben* and *cat* key functions.^80^ The complex nature of *ortho*-cleavage pathway gene expression underlines the importance of orthogonal expression to fine-tune biochemical functions for stable catalytic performance. Integration of a constitutive promoter upstream of *benD* or *catA-II* caused a significantly enhanced 3-FBz consumption, and these elements could have also boosted expression of benzoate transporter genes downstream. On the other hand, regulation of the transporter-encoding genes *benK*, *benE-II*, or *nicP-I* by *Pm*(*BCD10*) resulted in decreased 3-FBz consumption rates. These observations suggest a rate-limiting role of substrate transport in this set of strains, which is a typical bottleneck in industrial processes involving lignin-derived aromatic substrates.^98^

*P*. *putida* co-consumes glucose and benzoate at similar *q*_S_,^99^^,^^100^ with the expression of genes involved in substrate utilization controlled by Crc/Hfq.^101^, ^102^, ^103^ In this study, inactivation of *crc* without replacing the native P_*ben*_ promoter significantly increased *q*_S_ compared with a strain harboring P_*tac*_ → *benABC*. However, the substantial increase in 3-FBz uptake was not counteracted by a sufficient catechol 1,2-dioxygenase activity, leading to high 3-FC accumulation. Deletion of *crc* was found to be beneficial for improving the production of *cc*MA from lignocellulosic feedstocks.^104^ However, the assimilation of halogenated compounds clearly requires alternative regulatory regimes to balance biochemical activities. In our experiments, eliminating *crc* in several engineered strain backgrounds hindered the cells' ability to adapt to changing culture conditions, and Δ*crc* strains exhibited two distinct metabolic states in temporally separated phases during bioconversion (data not shown). Rather than merely establishing a hierarchy for assimilation of different substrates, Crc coordinates conflicting metabolic fluxes (e.g., glycolytic and gluconeogenic regimes).^105^ Blocking Crc-mediated regulation has been shown to alter the consumption of available carbon compounds in a complex medium, resulting in metabolite overflow and, consequently, inefficient growth.^106^ In particular, glucose consumption was enhanced during early exponential growth, significantly decreasing in the late exponential phase. These effects are also relevant under bioconversion conditions, whereby a non-native substrate (3-FBz) is supplied along sugars, and provide an explanation for the low glucose consumption of strain PMP1053d pre-grown in a rich medium. Thus, the interplay between the nutrients available for cell growth and the substrate for bioconversion (orchestrated by Crc) had to be optimized toward balanced 2-FMA biosynthesis.

### Combinatorial pathway balancing as an enabling technology to exploit the untapped metabolic diversity of *Pseudomonas* cell factories

The chemical landscape of bioproduction has been largely restricted to a relatively narrow range of molecules that have made their way to commercialization.^107^ In this sense, the vast majority of studies reporting bioproduction of novel molecules rely on the implementation of entirely synthetic biochemical routes,^108^ either *in vitro*^109^ or by engineering the production pathway in a microbial host.^110^ In this study we have adopted an entirely different approach, whereby the versatility of the rich native metabolism of *Pseudomonas* species^111^ has been harnessed for biocatalysis. Here, reshuffling existing elements of the bacterial biochemical network—rather than tackling *de novo* engineering efforts—was achieved at both the local and global levels of transcriptional regulation by implementing multiple rounds of the “design-build-test-learn” cycle of synthetic metabolic engineering. This approach enabled catalytic access to new-to-industry molecules without disturbing the extant metabolic architecture of the host. The value of this strategy was illustrated by the stoichiometric conversion of all fluorinated substrate into 2-FMA, the target product, at both maximum theoretical yield and isomeric purity—which is impossible when using traditional chemical synthesis protocols. We expect that this type of “inspired-by-Nature” blueprint to synthetic metabolism will broaden the catalytic power of bacterial cell factories even further, mediating a true transition to bio-based production of compounds that were not accessible thus far.

## Experimental procedures

### Resource availability

#### Lead contact

Further information and requests for resources should be directed to and will be fulfilled by the lead contact, Pablo I. Nikel (pabnik@biosustain.dtu.dk).

#### Materials availability

All materials generated in this study are available for research purposes from the lead contact.

### Bacterial strains and culture conditions

The bacterial strains employed in this study are listed in Table S3. *E*. *coli* and *P*. *putida* were incubated at 37°C and 30°C, respectively. For cell propagation and storage, routine cloning procedures, and during genome engineering manipulations, cells were grown in lysogeny broth (LB) medium (10 g L^−1^ tryptone, 5 g L^−1^ yeast extract, and 10 g L^−1^ NaCl). Cultures were performed using either 50-mL tubes with 5–10 mL of medium, or in 500-mL Erlenmeyer flasks capped with cellulose plugs (Carl Roth, Karlsruhe, Germany) containing 50 mL of medium. All liquid cultures were agitated at 250 rpm (MaxQ8000 incubator; Thermo Fisher Scientific, Waltham, MA, USA). Solid culture media contained 15 g L^−1^ agar. Kanamycin (Km), gentamicin (Gm), or streptomycin (Sm) were added when required at 50 μg mL^−1^, 10 μg mL^−1^, and 50 μg mL^−1^, respectively. For phenotypic characterizations in microtiter plates as well as shaken-flask fermentations, *P*. *putida* was pre-grown in LB medium, and the experiments were performed in synthetic DBM medium^112^ buffered with 5 g L^−1^ 3-(*N*-morpholino)propanesulfonic acid (MOPS) at pH 7.0 and supplemented with different carbon compounds as explained in the text. LB pre-cultures were harvested by centrifugation at 4,000 × *g* for 10 min, washed with DBM medium without any carbon substrate, and resuspended in the final medium of the experiment at the desired OD_600_. Cell growth was monitored by measuring the absorbance at 630 nm (*A*_630_) (for plate-reader experiments, with ELx808; BioTek Instruments, Winooski, VT, USA) or 600 nm (for shaken-flask experiments). The OD_600_ was estimated from plate-reader *A*_630_ values by multiplying the values by correlation factors previously determined for the employed microtiter plate readers and spectrophotometers. For calculation of quantitative cell performance parameters for shaken-flask experiments, biomass concentrations (cell dry weight, g_CDW_ L^−1^) were derived from OD_600_ measurements with a correlation factor of 0.35, previously determined for the spectrophotometer employed with exponentially growing *P*. *putida* KT2440. Comparative phenotypical characterizations and quantifications of green fluorescence for bioreporter strains were performed in 96-well plates in a Synergy H1 plate reader (BioTek Instruments). In this case, LB pre-cultures were diluted 1:100 in the respective screening medium (DBM medium supplemented with various organic compounds). Fluorescence was measured at an excitation wavelength of 488 nm and an emission wavelength of 588 nm, with the gain set to 60.

### Cloning procedures and plasmid construction

All plasmids used in this work are listed in Table S4. Uracil-excision (*USER*) cloning was used for the construction of all plasmids.^113^ The AMUSER tool was employed for the design of oligonucleotides.^114^ All genetic manipulations followed protocols published previously.^115^, ^116^, ^117^, ^118^, ^119^ *E*. *coli* DH5α λ*pir* was employed for all cloning purposes. Chemically competent *E*. *coli* cells were prepared and transformed with plasmids according to well-established protocols.^120^

### Metabolite analysis by HPLC

Supernatants were obtained via centrifugation of culture broths for 2 min at 13,000 × *g*. 2-FMA, *cc*MA, catechol, 3-FC, 4-FC, 2-chlorobenzoate, 3-chlorobenzoate, 4-chlorobenzoate, 2-FBz, and 3-FBz were quantified using a Dionex 3000 HPLC system equipped with a Zorbax Eclipse Plus C18 column (Agilent Technologies, Santa Clara, CA, USA) heated to 30°C and a guard column from Phenomenex. Separation was achieved with a mobile phase consisting of 0.05% (w/v) acetic acid and varying amounts of acetonitrile. The total runtime per sample was 8.3 min (with a separation time of 8.0 min), during which the fraction of acetonitrile was increased from 1% to 3% (v/v) within the first 3 min, followed by a steady increase to 20% (v/v) within 12 s and a further steady increase to 75% (v/v) within 4 min. From 7.2 to 7.5 min, the acetonitrile concentration was held at 75% (v/v) and subsequently reduced to 1% (v/v) within 18 s. The column was then equilibrated again at 1% (v/v) acetonitrile for further 30 s before injecting the next sample. The flow rate was set to 1 mL min^−1^, and the injection volume was 0.75 μL. After elution, the compounds were detected in the UV spectrum at 210 nm, 240 nm, 280 nm, and 300 nm. HPLC data were processed using the Chromeleon 7.1.3 software (Thermo Fisher Scientific), and compound concentrations were calculated from peak areas using calibration curves with five different standard concentrations. An authentic 2-FMA standard was chemically synthesized by Ambinter (Orléans, France). In this case, a solution of 32% (w/v) peracetic acid (36.0 g), glacial acetic acid (10 g), and ferric ammonium citrate (20 mg) was placed in a round flask (100 mL total volume). To the magnetically stirred mixture was added a solution of 3-FC (5.5 g) in glacial acetic acid (15 g) over 2 h at room temperature. After complete addition, the reaction mixture was stirred for an additional 24 h at room temperature. The resulting suspension was concentrated in vacuum without heating to a final volume of approximately 40 mL. After the solution was cooled to 0°C–4°C, the racemic product was collected by filtration, washed two times with ice-cold water, and dried in vacuum. The yield of the reaction sequence was 36.7%.

### Fluorometabolite analysis by ^19^F-NMR

^19^F-NMR spectra were acquired on a Bruker Avance III-HD spectrometer operating at a ^19^F frequency of 752.75 MHz (*B*_0_ = 18.8 T). The spectrometer was equipped with a TCI CryoProbe, and all measurements were made at 25°C. All 512 transient scans were acquired with an interscan delay of 5.6 s (0.6 s acquisition time followed by 5 s of recovery delay), tested to provide quantitative signal intensities for the relevant fluorinated species. Samples (500 μL) were mixed with 50 μL of D_2_O (Sigma-Aldrich, 99.99%) for locking and shimming. ^19^F chemical shifts are reported relative to CFCl_3_ (*δ*_19F_ = 0.0 ppm) using the lock signal of D_2_O as a secondary reference.^121^ All chemical shifts are reported in ppm.

### Data analysis

Data handling and calculations were performed in Microsoft Excel (2016) and OriginPro 2021 (OriginLab). Figures and illustrations were created in OriginPro (2021) and Adobe Illustrator 2020. Geneious Prime 2021.1.1 (Biomatters) served as a database for DNA sequences to design plasmids and constructs and to analyze Sanger sequencing results. Maximum exponential growth rates (*μ*_max_) were determined by Gaussian process regression using the Python-based tool deODorizer.^122^ The prediction of translation initiation strengths for 5′ UTR mRNA sequences was performed using the online RBS Calculator v2.1.^123^ The results are given in arbitrary units (au) on the RBS Calculator scale, representing the relative strength of translation. Specific fluorobenzoate consumption rates (*q*_S_) and 2-FMA production rates (*q*_P_) were determined over the timeframes of fermentations in which changes in the extracellular concentrations of 3-FBz and 2-FMA were detectable with the following equations:

(Equation 1) $$q_{\text{S}}=\frac{1}{\bar{X}}\frac{\Delta S}{\Delta t}\text{,}$$

(Equation 2) $$q_{\text{P}}=\frac{1}{\bar{X}}\frac{\Delta P}{\Delta t}\text{,}$$

where *q*_S_ is the biomass-specific substrate consumption rate (mmol g_CDW_^−1^ h^−1^), $\bar{X}$ is the average biomass concentration between two sampling timepoints (g_CDW_ L^−1^), Δ*S* is the difference in substrate concentration between two sampling timepoints (mM), Δ*t* is the time between two sampling points (h), *q*_P_ is the biomass-specific product formation rate (mmol g_CDW_^−1^ h^−1^), and Δ*P* is the difference in product concentration between two sampling timepoints (mM).

The *q*_S_ and *q*_P_ values displayed in Figure 6 and Table S5 are averages of the values determined individually for three biological replicates. The transcription and translation initiation strengths of various expression systems tested in reporter experiments were determined via linear regression of fluorescence-over-OD_600_ plots in OriginPro (2021). The identified slope values were normalized by dividing them by the expression strength of the P_*tac*_ promoter, used as a reference.

## Data Availability

This study did not generate any datasets or code.

## Supplemental information

Document S1. Figures S1–S4 and Tables S1–S5

Data S1. Data_per_Figure.xlsx

Document S2. Article plus supplemental information
