# Supplementary material for: Combinatorial pathway balancing provides biosynthetic access to 2-fluoro-cis,cis-muconate in engineered Pseudomonas putida
Source: Chem Catal. 2021 Nov 18;1(6):1234–59. doi: 10.1016/j.checat.2021.09.002 (PMC8711041; doi:10.1016/j.checat.2021.09.002)
Supplement: Document S1. Figures S1–S4 and Tables S1–S5 [file mmc1.pdf]

Chem Catalysis, Volume *1*

Supplemental information

Combinatorial pathway balancing provides biosynthetic access to 2-fluoro-*cis*,*cis*-muconate in engineered *Pseudomonas putida*

Nicolas T. Wirth and Pablo I. Nikel

SUPPLEMENTARY TABLES

Table S1. Genes encoding enzymes involved in processing halogenated benzoates in *P. knackmussii* and their homologs in *P. putida* KT2440 (or in the catabolic pWW0 TOL plasmid of strain mt-2).

Table S2. Specific growth rates of selected strains used in this study.

Table S3. Bacterial strains used in this study.

Table S4. Plasmids used in this study.

Table S5. Performance parameters for selected strains used in this study in the bioconversion of 10 mM 3-FBz.

SUPPLEMENTARY FIGURES

Figure S1. Physiological response to fluorinated and nonfluorinated ortho-cleavage metabolites.

Figure S2. Quantification of (fluoro)metabolites via HPLC.

Figure S3. Transcriptome analysis of genes within the *ben* and *cat* clusters.

Figure S4. Bioconversion performance of second-generation strains with altered transporter expression.

Table S1. Genes encoding enzymes involved in processing halogenated benzoates in *P. knackmussii* and their homologs in *P. putida* KT2440 (or in the catabolic pWW0 TOL plasmid of strain mt-2).

P. knackmussii gene	*P. putida* KT2440 protein with highest homology │ % identity	pWW0 protein with highest homology │ % identity	Encoded function
PKB_1739	CatA-II (*PP_3166*) │ 78.3%<br>CatA (*PP_3713*) │ 74.1%	none	Catechol 1,2-dioxygenase
PKB_2098	BenR (*PP_3159*) │ 72.6%	XylS │ 58.7%	Transcriptional regulator (XylS homolog)
PKB_2100	BenA (*PP_3161*) │ 86.7%	XylX │ 71.7%	Benzoate 1,2-dioxygenase subunit alpha
PKB_2101	BenB (*PP_3162*) │ 83.9%	XylY │ 77.2%	Benzoate 1,2-dioxygenase subunit beta
PKB_2102	BenC (*PP_3163*) │ 84.8%	XylZ │ 85.2%	Benzoate 1,2-dioxygenase electron transfer component
PKB_2103	BenD (*PP_3164*) │ 79.7%	XylL │ 77.0%	1,2-Dihydroxybenzoate dehydrogenase
PKB_2104	CatR (*PP_3716*) │ 81.5%	none	HTH-type transcriptional regulator
PKB_2105	CatB (*PP_3715*) │ 85%	none	Muconate cycloisomerase
PKB_2106	CatC (*PP_3714*) │ 92.7%	none	Muconolactone δ-isomerase
PKB_2107	CatA-II (*PP_3166*) │ 80.5%<br>CatA (*PP_3713*) │ 75.2%	none	catechol 1,2-dioxygenase
PKB_3272	YnfL (*PP_5071*) │ 32.6%<br>CatR (*PP_3716*) │ 33.0%	none	LysR family transcriptional regulator
PKB_3273	CatA (*PP_3713*) │ 31.3%<br>CatA-II (*PP_3166*) │ 29.1%	none	Catechol 1,2-dioxygenase
PKB_3274	CatB (*PP_3715*) │ 42.0%	none	Muconate cycloisomerase
PKB_3621 (identical to *PKB_3272*)	YnfL (*PP_5071*) │ 32.6%<br>CatR (*PP_3716*) │ 33.0%	none	LysR family transcriptional regulator
PKB_3622 (identical to *PKB_3273*)	CatA (*PP_3713*) │ 31.3%<br>CatA-II (*PP_3166*) │ 29.1%	none	Catechol 1,2-dioxygenase
PKB_3623 (identical to *PKB_3274*)	CatB (*PP_3715*) │ 42.0%	none	Muconate and cycloisomerase

Table rows are shaded to indicate the respective genes' location within separate clusters in the chromosome of *P. knackmussii*. Homology values are based on the amino acid sequences of proteins encoded by the listed genes.

Table S2. Specific growth rates of selected strains used in this study.

Medium additives	Maximum specific growth rate, μ_{max} (h^{-1}), for strain:			
	P. putida KT2440	*P. knackmussii*	PMP1021/ pSEVA228	PMP1053
30 mM glucose	1.04 ± 0.09	0.40 ± 0.08	0.76 ± 0.16	0.91 ± 0.12
30 mM Bz (+ 0.5 mM 3-*m*Bz)	0.73 ± 0.14	no growth	0.16 ± 0.09	0.39 ± 0.03
30 mM 2-FBz	no growth	no growth	n.d.	no growth
30 mM 3-FBz	no growth	no growth	n.d.	no growth
30 mM 4-FBz	no growth	no growth	n.d.	no growth
30 mM glucose, 1 mM catechol	0.83 ± 0.17	0.36 ± 0.06	0.83 ± 0.13	n.d.
30 mM glucose, 2 mM catechol	0.78 ± 0.14	0.32 ± 0.01	0.89 ± 0.11	n.d.
30 mM glucose, 1 mM 3-FC	0.63 ± 0.10	0.22 ± 0.05	0.49 ± 0.03	n.d.
30 mM glucose, 2 mM 3-FC	0.37 ± 0.01	0.12 ± 0.02	0.22 ± 0.02	no growth
30 mM glucose, 5 mM 3-FC	no growth	no growth	no growth	no growth
30 mM glucose, 1 mM 4-FC	0.60 ± 0.11	0.24 ± 0.03	0.63 ± 0.19	n.d.
30 mM glucose, 2 mM 4-FC	0.76 ± 0.08	0.30 ± 0.01	0.50 ± 0.05	0.76 ± 0.13
30 mM glucose, 5 mM 4-FC	no growth	no growth	no growth	no growth
30 mM glucose, 10 mM 2-FBz	0.65 ± 0.13	0.26 ± 0.02	n.d.	n.d.
30 mM glucose, 15 mM 2-FBz	0.61 ± 0.12	0.22 ± 0.03	n.d.	n.d.
30 mM glucose, 20 mM 2-FBz	0.56 ± 0.14	no growth	n.d.	n.d.
30 mM glucose, 5 mM 3-FBz	0.39 ± 0.19	0.05 ± 0.01	0.39 ± 0.06	n.d.
30 mM glucose, 10 mM 3-FBz	0.14 ± 0.08	no growth	0.38 ± 0.04	0.45 ± 0.02
30 mM glucose, 15 mM 3-FBz	no growth	no growth	0.36 ± 0.07	0.45 ± 0.03
30 mM glucose, 20 mM 3-FBz	no growth	no growth	0.35 ± 0.08	0.46 ± 0.02
30 mM glucose, 30 mM 3-FBz	no growth	no growth	n.d.	0.24 ± 0.04
30 mM glucose, 40 mM 3-FBz	no growth	no growth	n.d.	0.23 ± 0.05
30 mM glucose, 50 mM 3-FBz	no growth	no growth	n.d.	0.20 ± 0.04
30 mM glucose, 10 mM 2-FMA	0.98 ± 0.16	n.d.	n.d.	0.98 ± 0.15
30 mM glucose, 15 mM 2-FMA	0.66 ± 0.04	n.d.	n.d.	0.56 ± 0.05
30 mM glucose, 20 mM 2-FMA	no growth	n.d.	n.d.	no growth

The experiments were performed in 96-well microtiter plates with each well containing 200 µL of DBM medium buffered with 5 g L^{-1} MOPS and varying concentrations of carbon sources and (fluoro)metabolites involved in the bioconversion process. Maximum specific growth rates were determined by Gaussian process regression. Each experiment was performed in three biological replicates. Growth rates are given as average values ± standard deviation. *n.d.*, not determined.

Table S3. Bacterial strains used in this study.

Strain	Genotype / Relevant characteristics	Reference or source
Escherichia coli		
DH5α λ*pir*	Cloning host; F⁻ λ⁻ *endA1 glnX44*(AS) *thiE1 recA1 relA1 spoT1 gyrA96*(Nal^R) *rfbC1 deoR nupG* Φ80(*lacZ*ΔM15) Δ(*argF-lac*)*U169 hsdR17*(r_K^- m_K^+), λ pir lysogen. NCBI Taxonomy ID: 668369	Platt et al. [1]
Pseudomonas		
P. putida KT2440	Wild-type strain, derived from *P. putida* mt-2,[2] cured of the TOL plasmid pWW0. NCBI Taxonomy ID: 160488	Bagdasarian et al. [3]
P. knackmussii	Wild-type strain, haloaromatic degrader. NCBI Taxonomy ID: 65741	Stolz et al. [4]
PMP1000	P_{tac}→*benABC* *P. putida* KT2440 with the chromosomal *benABC* cluster under control of the constitutive P_{tac} promoter.[65] The native, validated 5'-UTR of *benA* was kept unchanged,[66] providing a predicted translation rate of 220.	This study
PMP0100	P_{tac}→*benD* *P. putida* KT2440 with *benD* under control of the constitutive P_{tac} promoter. The native 28 bp upstream of *benD* were kept unchanged, providing a translation initiation sequence with a predicted translation rate of 818.	This study
PMP0010	P_{EM7}→*catA* *P. putida* KT2440 with the constitutive P_{EM7} promoter chromosomally inserted upstream of *catA*. The native 29 bp upstream of *catA* were kept unchanged, providing a translation initiation sequence with a predicted translation rate of 3,211.	This study
PMP0000a	Δ*catBC* *P. putida* KT2440 with both *catB* and *catC* deleted.	This study
PMP0000b	Δ*catBC::catA* *P. putida* KT2440 with the genes *catB* and *catC* replaced with an additional copy of *catA*. The predicted translation rate of *catA* with the *catB* 5'-UTR is 1,934.	This study
PMP0000c	Δ*catBC::catA-II* *P. putida* KT2440 with *catB* and *catC* replaced with an additional copy of *catA-II*. The predicted translation rate of *catA-II* with the *catB* 5'-UTR is 503.	This study
PMP1000a	P_{tac}→*benABC* Δ*catBC*	This study
PMP0100a	P_{tac}→*benD* Δ*catBC*	This study
PMP0100b	P_{tac}→*benD* Δ*catBC::catA*	This study
PMP0100c	P_{tac}→*benD* Δ*catBC::catA-II*	This study
PMP0020	*Pm(BCD10)*→*catA* *P. putida* KT2440 with *catA* under control of the inducible *Pm* promoter and the translational coupling sequence *BCD10*[57] with predicted translation strengths of 28,177 (SD1) and 331 (SD2).	This study
PMP0001	*Pm(BCD10)*→*catA-II* *P. putida* KT2440 with *catA* under control of the inducible *Pm* promoter and the translational coupling sequence *BCD10* with predicted translation strengths of 28,177 (SD1) and 134 (SD2).	This study
PMP0021	*Pm(BCD10)*→*catA Pm(BCD10)*→*catA-II*	This study
PMP1021	P_{tac}→*benABC Pm(BCD10)*→*catA Pm(BCD10)*→*catA-II*	This study
PMP1041	P_{tac}→*benABC xylS/*$Pm_{(ML1\text{-}17)}$*(BCD10)*→*catA Pm(BCD10)*→*catA-II* Strain PMP1011 with *xylS* and its native regulatory sequences chromosomally integrated upstream of and encoded on the opposite DNA strand as *catA*, which is controlled by the inducible *Pm* promoter variant ML1-17.[13]	This study
PMP1021e	P_{tac}→*benABC Pm(BCD10)*→*catA Pm(BCD10)*→*catA-II* $Pm_{(ML1\text{-}17)}$*(BCD10)*→*nicP-I* Strain PMP1022 with *nicP-I* (*benF*, a porin-like protein) under control of the inducible *Pm* promoter variant ML1-17 and the translational coupling sequence *BCD10* with predicted translation strengths of 31,045 (SD1) and 72 (SD2).	This study
PMP1021f	P_{tac}→*benABC Pm(BCD10)*→*catA* *Pm(BCD10)*→*catA-II* $Pm_{(ML1\text{-}17)}$→*benE-II* Strain PMP1022 with *benE-II* (benzoate transporter) under control of the inducible *Pm* promoter variant ML1-17 and the translational coupling sequence *BCD10* with predicted translation strengths of 31,045 (SD1) and 1,594 (SD2).	This study
PMP1021g	P_{tac}→*benABC Pm(BCD10)*→*catA Pm(BCD10)*→*catA-II* $Pm_{(ML1\text{-}17)}$→*benK* Strain PMP1022 with *benK* (benzoate transporter) under control of the inducible *Pm* promoter variant ML1-17 and the translational coupling sequence *BCD10* with predicted translation strengths of 31,045 (SD1) and 541 (SD2).	This study
PMP1030	P_{tac}→*benABC Pm(BCD2)*→*catA* Chromosomal *benABC* gene cluster placed under the control of the constitutive P_{tac} promoter. Chromosomal *catA* under control of the inducible *Pm* promoter and the translational coupling sequence *BCD2*[12] with predicted translation strengths of 28,177 (SD1) and 18,908 (SD2).	This study
PMP1002	P_{tac}→*benABC Pm(BCD2)*→*catA-II* Chromosomal *benABC* gene cluster under control of the constitutive P_{tac} promoter. Chromosomal *catA-II* under control of the inducible *Pm* promoter and the translational coupling sequence *BCD2* with predicted translation strengths of 28,177 (SD1) and 7,687 (SD2).	This study
PMP1023	P_{tac}→*benABC Pm(BCD10)*→*catA* P_{14b}*(BCD10)*→*catA-II*	This study

	Chromosomal *catA-II* under the transcriptional control of the constitutive promoter P_{14b}.[14]	
PMP1053	P_{tac}→*benABC* P_{14b}*(BCD10)*→*catA* P_{14b}*(BCD10)*→*catA-II*<br>Chromosomal *catA* and *catA-II* under the transcriptional control of the constitutive promoter P_{14b}.	This study
PMP1053d	P_{tac}→*benABC* P_{14b}*(BCD10)*→*catA* P_{14b}*(BCD10)*→*catA-II* Δ*crc*	This study
PMP2053	P_{tac}*(BCD10)*→*benABC* P_{14b}*(BCD10)*→*catA* P_{14b}*(BCD10)*→*catA-II*	This study
PMP1024	P_{tac}→*benABC Pm(BCD10)*→*catA* P_{14g}→*xylS/Pm(BCD10)*→*catA-II*<br>Chromosomal *catA-II* under the transcriptional control of a XylS/*Pm* element, with *xylS* chromosomally integrated upstream of *catA-II* under control of P_{14g} and a synthetic translation initiation sequence with a translation rate of 719,862.	This study

A graphical representation of the strain nomenclature is provided in Figure 3A. Translation initiation rates were determined by using the online *RBS Calculator* 2.1.

Table S4. Plasmids used in this study.

Plasmid	Relevant characteristics [a]	Reference or source
pGNW2	Suicide vector used for genetic manipulations in Gram-negative bacteria; *oriT, traJ, lacZα, ori*(R6K), P_{14g}*(BCD2)→msfGFP;* Km^R	Wirth et al. [5]
pSNW2	Derivative of vector pGNW2 with the translation initiation sequence of *msfGFP* replaced by the very strong translational coupling sequence *BCD2*	Volke et al. [6]
pSEVA628S	Helper plasmid; *oriV*(RK2), *xylS*, *Pm→I-SceI*; Gm^R	Silva-Rocha et al. [7]
pSEVA627M	*oriV*(RK2), *msfGFP*, *oriT*, Gm^R	Silva-Rocha *et al.* [7]
pSEVA228	Plasmid used to supply XylS for chromosomal *Pm* promoters; *oriV*(RK2), *xylS*, *Pm*, Km^R	Martínez-García et al. [8]
pSEVA228.2	Plasmid used to supply $XylS^{Thr45}$ (XylS.2) for chromosomal *Pm* promoters; *oriV*(RK2)*, xylS.2*, *Pm*, Km^R	Ramos et al. [9]
pQURE6·H	Conditionally-replicating vector; derivative of vector pJBSD1 carrying XylS/*Pm→I-SceI* and P_{14g}*(BCD2)→mRFP*; Gm^R	Volke et al. [10]
pGNW2·P_{tac}*→benABC*	Derivative of pGNW2 carrying homology arms (HAs) to replace the native P_{ben} promoter upstream of *benA* with the P_{tac} promoter sequence in *P. putida* KT2440	This study<br>P_{tac}: de Boer et al. [11]
pGNW2·P_{tac}*→benD*	Derivative of pGNW2 carrying HAs to insert the P_{tac} promoter sequence upstream of *benD* in *P. putida* KT2440	This study
pGNW2·P_{EM7}*→catA*	Derivative of pGNW2 carrying HAs to insert the P_{EM7} promoter sequence upstream of *catA* in *P. putida* KT2440	This study
pGNW2·*Pm(BCD10)→catA*	Derivative of pGNW2 carrying HAs to insert the *Pm* promoter and the translational coupling sequence *BCD10* upstream of *catA* in *P. putida* KT2440	This study<br>*BCD10*: Mutalik et al. [12]
pGNW2·*Pm(BCD2)→catA*	Derivative of pGNW2 carrying HAs to insert the *Pm* promoter and the translational coupling sequence *BCD2* upstream of *catA* in *P. putida* KT2440	This study<br>*BCD2*: Mutalik *et al.* [12]
pGNW2·*xylS*/$Pm_{(ML1-17)}$*(BCD10)→ catA*	Derivative of pGNW2 carrying HAs to insert the *xylS* gene with its native regulatory sequences, the *Pm* promoter variant *ML1-17*, and the translational coupling sequence *BCD10* upstream of *catA* in *P. putida* KT2440	This study<br>$Pm_{(ML1-17)}$: Bakke et al. [13]
pGNW2·P_{14g}*(BCD10)→xylS/Pm (BCD10)→catA*	Derivative of pGNW2 carrying HAs to insert the *xylS* gene under the control of P_{14g} and *BCD10*, the *Pm* promoter, and the translational coupling sequence *BCD10* upstream of *catA* in *P. putida* KT2440	This study
pGNW2·*Pm(BCD10)→catA-II*	Derivative of pGNW2 carrying HAs to insert the *Pm* promoter and the translational coupling sequence *BCD10* upstream of *catA-II* in *P. putida* KT2440	This study
pGNW2·*Pm(BCD2)→catA-II*	Derivative of pGNW2 carrying HAs to insert the *Pm* promoter and the translational coupling sequence *BCD2* upstream of *catA-II* in *P. putida* KT2440	This study
pSNW2·*Pm(BCD10)→nicP-I*	Derivative of pGNW2 carrying HAs to insert the *Pm* promoter and the translational coupling sequence *BCD10* upstream of *nicP-I* in *P. putida* KT2440	This study
pSNW2·*Pm(BCD10)→benE-II*	Derivative of pGNW2 carrying HAs to insert the *Pm* promoter and the translational coupling sequence *BCD10* upstream of *benE-II* in *P. putida* KT2440	This study
pSNW2·*Pm(BCD10)→benK*	Derivative of pGNW2 carrying HAs to insert the *Pm* promoter and the translational coupling sequence *BCD10* upstream of *benK* in *P. putida* KT2440	This study
pGNW2·Δ*crc*	Derivative of pGNW2 carrying HAs to delete *crc* in *P. putida* KT2440	This study
pGNW·Δ*catBC*	Derivative of pGNW2 carrying HAs to delete *catBC* in *P. putida* KT2440	This study
pGNW·Δ*catBC::catA*	Derivative of pGNW2 carrying HAs to delete *catBC* in *P. putida* KT2440 replace it with an additional copy of *catA*	This study
pGNW·Δ*catBC::catA-II*	Derivative of pGNW2 carrying HAs to delete *catBC* in *P. putida* KT2440 replace it with an additional copy of *catA-II*	This study
pGNW2·P_{tac}*(BCD10)→benABC*	Derivative of pGNW2 carrying homology arms (HAs) to replace the native P_{ben} promoter upstream of *benA* with the P_{tac} promoter sequence, and the native 5'-UTR with the translational coupling sequence *BCD10* in *P. putida* KT2440.	This study
pGNW2·P_{14b}*(BCD10)→catA*	Derivative of pGNW2 carrying homology arms (HAs) to insert the constitutive P_{14b} promoter and the translational coupling sequence *BCD10* upstream of *catA* in *P. putida* KT2440.	This study<br>P_{14b}: Zobel et al. [14]
pGNW2·P_{14b}*(BCD10)→catA-II*	Derivative of pGNW2 carrying homology arms (HAs) to insert the constitutive P_{14b} promoter and the translational coupling sequence *BCD10* upstream of *catA-II* in *P. putida* KT2440.	This study
pGNW2·P_{14g}*→xylS/Pm(BCD2)→ catA-II*	Derivative of pGNW2 carrying homology arms (HAs) to insert the *Pm* promoter and the translational coupling sequence *BCD10,* as well as the	This study

	gene sequence of *xylS* under the control of the strong, constitutive promoter P_{14g}, upstream of *catA-II* in *P. putida* KT2440.	
pS628*(BCD1)→msfGFP*	*oriV*(RK2), *xylS*, *Pm(BCD1)→msfGFP*; Gm^R	This study<br>*BCD1*: Mutalik *et al.* [12]
pS628*(BCD2)→ msfGFP*	*oriV*(RK2), *xylS*, *Pm(BCD2)→msfGFP*; Gm^R	This study
pS628*(BCD7)→ msfGFP*	*oriV*(RK2), *xylS*, *Pm(BCD7)→msfGFP*; Gm^R	This study<br>*BCD7*: Mutalik *et al.* [12]
pS628*(BCD10)*→msfGFP	*oriV*(RK2), *xylS*, *Pm(BCD10)→msfGFP*; Gm^R	This study
pS628$_{(ML1-17)}$*(BCD10)*→msfGFP	*oriV*(RK2), *xylS*, *Pm*$_{(ML1-17)}$*(BCD10)→msfGFP*; Gm^R	This study
pS62P_{tac}*(BCD10)*→msfGFP	*oriV*(RK2), P_{tac}*(BCD10)→msfGFP*; Gm^R	This study
pS62P_{EM7}*(BCD10)*→ msfGFP	*oriV*(RK2), P_{EM7}*(BCD10)→msfGFP*; Gm^R	This study
pS62P_{14g}*(BCD10)*→msfGFP	*oriV*(RK2), P_{14g}*(BCD10)→msfGFP*; Gm^R	This study<br>P_{14g}: Zobel *et al.* [14]
pS62P_{14d}*(BCD10)*→msfGFP	*oriV*(RK2), P_{14d}*(BCD10)→msfGFP*; Gm^R	This study<br>P_{14d}: Zobel *et al.* [14]
pS62P_{J23108}*(BCD10)*→msfGFP	*oriV*(RK2), P_{J23108}*(BCD10)→msfGFP*; Gm^R	This study
pS62P_{J23114}*(BCD10)*→msfGFP	*oriV*(RK2), P_{J23114}*(BCD10)→msfGFP*; Gm^R	This study
pS62P_{J23119}*(BCD10)*→msfGFP	*oriV*(RK2), P_{J23119}*(BCD10)→msfGFP*; Gm^R	This study
pS62P_{cat}*(BCD10)→msfGFP*	*oriV*(RK2), *catR*, P_{cat}*(BCD10)→msfGFP*, Gm^R	This study
pS62P_{ben}*(BCD10)→msfGFP*	*oriV*(RK2), *benR*, P_{ben}*(BCD10)→msfGFP*; Gm^R	This study
pS634·*PKB_1379*	*oriV*(pBBR1), *lacIq*, P_{trc}*→PKB_1379*, Gm^R	This study
pS634·*PKB_2107*	*oriV*(pBBR1), *lacIq*, P_{trc}*→PKB_2107*, Gm^R	This study
pS634·*PKB_3273*	*oriV*(pBBR1), *lacIq*, P_{trc}*→PKB_3273*, Gm^R	This study

Table S5. Performance parameters for selected strains used in this study in the bioconversion of 10 mM 3-FBz.

**Strain**	**μ_{max}** [h^{-1}]	**q_S** [mmol g_{CDW}^{-1} h^{-1}]	**q_P** [mmol g_{CDW}^{-1} h^{-1}]
P. putida KT2440	0.14 ± 0.08	0.18 ± 0.02 [a]	0.06 ± 0.03
PMP1021/pSEVA228	0.38 ± 0.04	1.02 ± 0.07	0.47 ± 0.10 [b]
PMP1021e/pSEVA228	0.29 ± 0.02	0.57 ± 0.01	0.32 ± 0.03 [b]
PMP1021f/pSEVA228	0.30 ± 0.01	0.84 ± 0.05	0.41 ± 0.03 [b]
PMP1021g/pSEVA228	0.27 ± 0.01	0.74 ± 0.01	0.36 ± 0.02 [b]
PMP1030/pSEVA228	0.32 ± 0.03	1.21 ± 0.11	0.17 ± 0.04
PMP1002/pSEVA228	0.39 ± 0.02	0.81 ± 0.01	0.38 ± 0.02 [b]
PMP1053	0.45 ± 0.02	1.24 ± 0.01	0.63 ± 0.04 [b]
PMP1053d	0.15 ± 0.01	1.67 ± 0.09 [a]	0.53 ± 0.02
PMP2053	0.41 ± 0.02	0.26 ± 0.01 [a]	0.13 ± 0.05 [b]
PMP1023/pSEVA228	0.29 ± 0.02	1.10 ± 0.12	0.43 ± 0.03 [b]
PMP1023/pSEVA228.2	0.25 ± 0.02	0.84 ± 0.01	0.36 ± 0.01 [b]
PMP1024	0.30 ± 0.01	0.95 ± 0.11	0.48 ± 0.02 [b]

To determine the biomass-specific 3-FBz uptake rates (q_S) and 2-FMA formation rates (q_P), the strains were cultured in Erlenmeyer flasks filled with 10% (v/v) DBM medium supplemented with 30 mM glucose and 10 mM 3-FBz. Maximum specific growth rates (μ_{max}) were determined in microtiter plate experiments in the same medium. [a] incomplete consumption of 3-FBz; [b] no detectable fluorocatechol at the end of the fermentation.

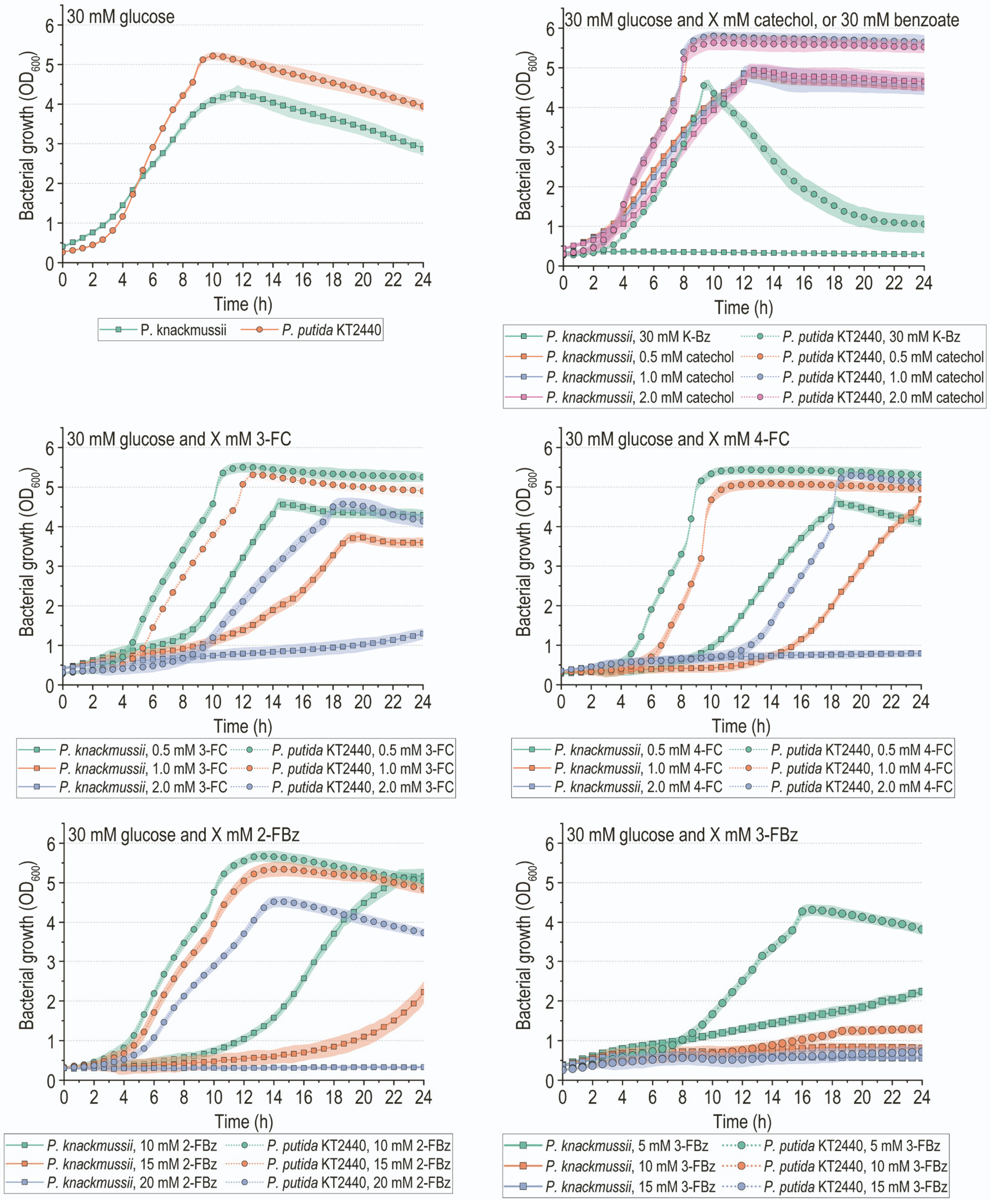

Figure S1. Physiological response to fluorinated and nonfluorinated *ortho*-cleavage metabolites. *P. knackmussii* and *P. putida* KT2440 were cultured in microtiter plates with 200 µL of DBM medium supplemented with 30 mM glucose or potassium benzoate (K-Bz) as the source of carbon and energy, as well as varying concentrations of metabolites involved in the bioconversion process. Error bars represent the standard deviations from three biological replicates.

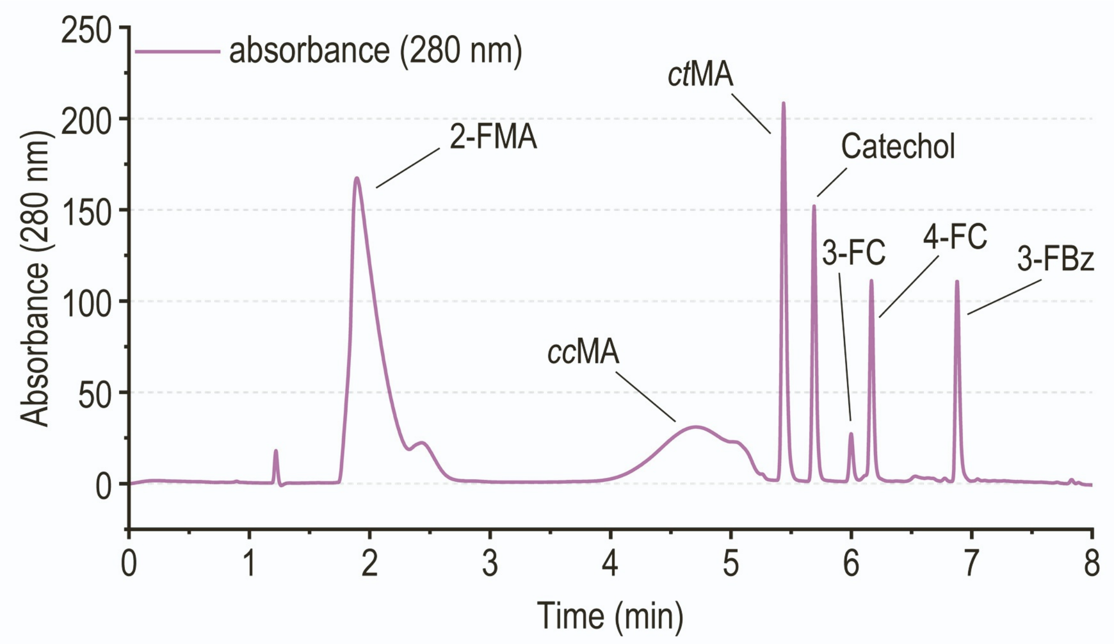

Figure S2. Quantification of (fluoro)metabolites *via* HPLC. The displayed chromatogram represents a calibration standard containing 1 mM of each compound with UV absorption measured at 280 nm.2-FBz eluted at a retention time of 6.4 min (not shown).

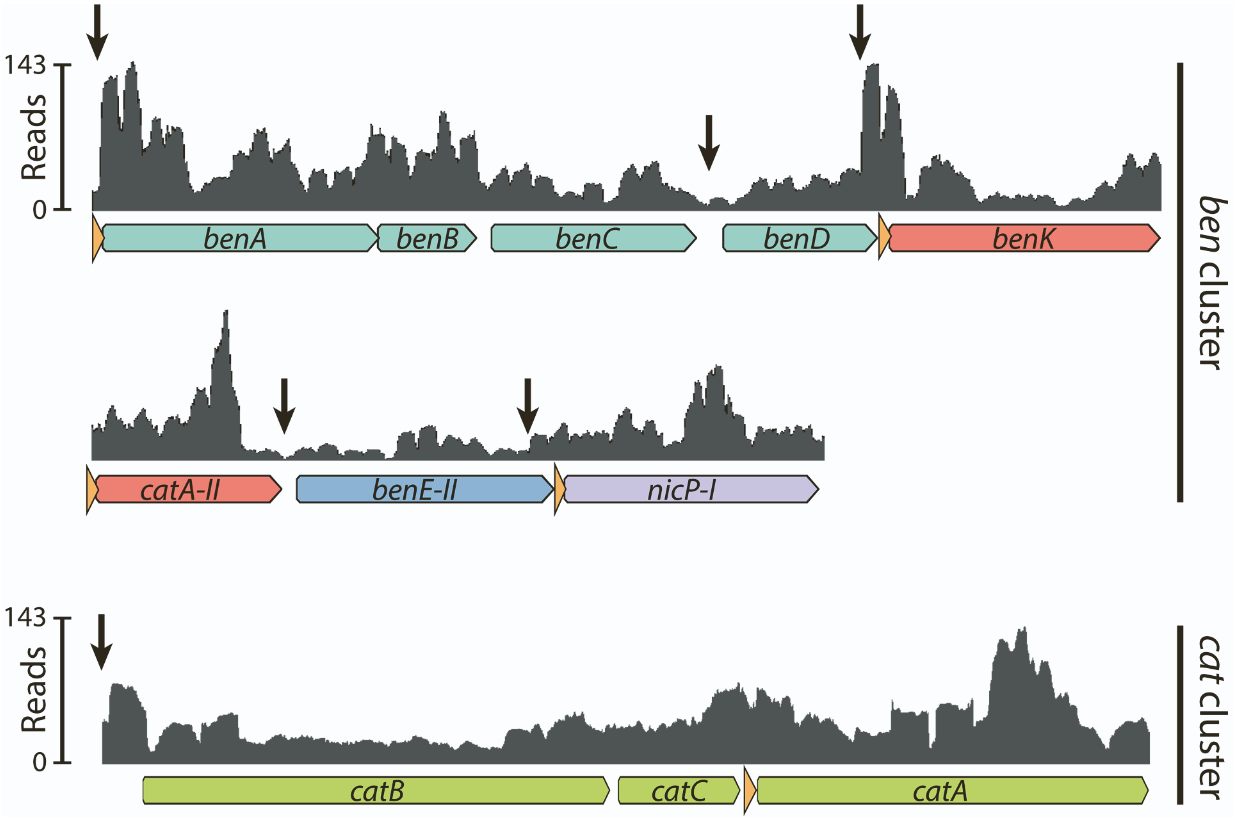

Figure S3. Transcriptome analysis of genes within the *ben* and *cat* clusters. Transcriptomic data published under various experimental conditions was pooled and mapped to the chromosome sequence of *P. putida* KT2440 (NCBI RefSeq NC_002947). Grey columns represent the read coverage for every nucleotide position. Orange triangles indicate identified Hfq recognition sequences. Black arrows highlight potential transcription start positions inferred from the course of coverage. The genes are drawn with colors indicating concerted expression as a transcription unit based on computational predictions on BioCyc.org.[15]

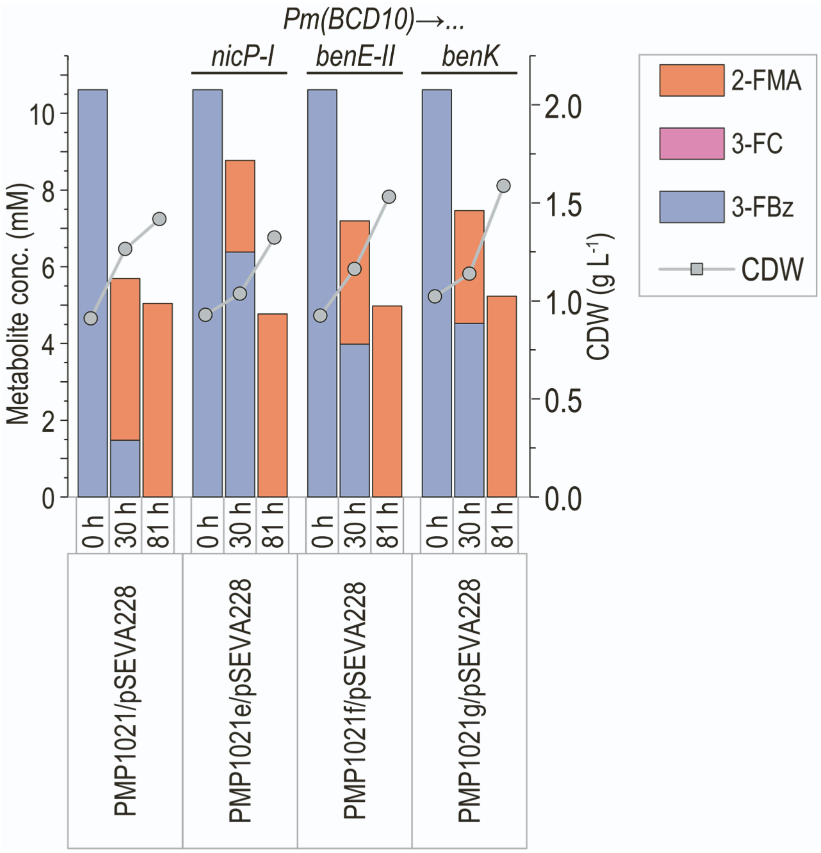

Figure S4. Bioconversion performance of *second-generation* strains with altered transporter expression. Strains were cultured in Erlenmeyer flasks filled with 10% (v/v) DBM medium supplemented with 30 mM glucose, 5 g L^{-1} MOPS, and 10 mM 3-FBz.

REFERENCES

1. Platt, R., Drescher, C., Park, S.K., and Phillips, G.J. (2000). Genetic system for reversible integration of DNA constructs and *lacZ* gene fusions into the *Escherichia coli* chromosome. Plasmid *43*, 12-23. 10.1006/plas.1999.1433.

2. Worsey, M.J., and Williams, P.A. (1975). Metabolism of toluene and xylenes by *Pseudomonas putida* (*arvilla*) mt-2: evidence for a new function of the TOL plasmid. J. Bacteriol. *124*, 7-13.

3. Bagdasarian, M., Lurz, R., Rückert, B., Franklin, F.C.H., Bagdasarian, M.M., Frey, J., and Timmis, K.N. (1981). Specific purpose plasmid cloning vectors. II. Broad host range, high copy number, RSF1010-derived vectors, and a host-vector system for gene cloning in *Pseudomonas*. Gene *16*, 237-247. 10.1016/0378-1119(81)90080-9.

4. Stolz, A., Busse, H.-J., and Kämpfer, P. (2007). *Pseudomonas knackmussii* sp. nov. Internat. J. Syst. Evol. Microbiol. *57*, 572-576. 10.1099/ijs.0.64761-0.

5. Wirth, N.T., Kozaeva, E., and Nikel, P.I. (2020). Accelerated genome engineering of *Pseudomonas putida* by I-*Sce*I—mediated recombination and CRISPR-Cas9 counterselection. Microb. Biotechnol. *13*, 233-249. 10.1111/1751-7915.13396.

6. Volke, D.C., Friis, L., Wirth, N.T., Turlin, J., and Nikel, P.I. (2020). Synthetic control of plasmid replication enables target- and self-curing of vectors and expedites genome engineering of *Pseudomonas putida*. Metab. Eng. Commun. *10*, e00126. 10.1016/j.mec.2020.e00126.

7. Silva-Rocha, R., Martínez-García, E., Calles, B., Chavarría, M., Arce-Rodríguez, A., de las Heras, A., Páez-Espino, A.D., Durante-Rodríguez, G., Kim, J., Nikel, P.I., et al. (2013). The Standard European Vector Architecture (SEVA): a coherent platform for the analysis and deployment of complex prokaryotic phenotypes. Nucleic Acids Res. *41*, D666-D675. 10.1093/nar/gks1119.

8. Martínez-García, E., Aparicio, T., Goñi-Moreno, A., Fraile, S., and de Lorenzo, V. (2015). SEVA 2.0: an update of the Standard European Vector Architecture for de-/re-construction of bacterial functionalities. Nucleic Acids Res. *43*, D1183-D1189. 10.1093/nar/gku1114.

9. Ramos, J.L., Michan, C., Rojo, F., Dwyer, D., and Timmis, K. (1990). Signal-regulator interactions, genetic analysis of the effector binding site of *xylS*, the benzoate-activated positive regulator of *Pseudomonas* TOL plasmid *meta*-cleavage pathway operon. J. Mol. Biol. *211*, 373-382. 10.1016/0022-2836(90)90358-S.

10. Volke, D.C., Turlin, J., Mol, V., and Nikel, P.I. (2020). Physical decoupling of XylS/*Pm* regulatory elements and conditional proteolysis enable precise control of gene expression in *Pseudomonas putida*. Microb. Biotechnol. *13*, 222-232. 10.1111/1751-7915.13383.

11. de Boer, H.A., Comstock, L.J., and Vasser, M. (1983). The *tac* promoter: a functional hybrid derived from the *trp* and *lac* promoters. Proc. Natl. Acad. Sci. USA *80*, 21-25. 10.1073/pnas.80.1.21.

12. Mutalik, V.K., Guimaraes, J.C., Cambray, G., Lam, C., Christoffersen, M.J., Mai, Q.A., Tran, A.B., Paull, M., Keasling, J.D., Arkin, A.P., and Endy, D. (2013). Precise and reliable gene expression via standard transcription and translation initiation elements. Nat. Methods *10*, 354-360. 10.1038/nmeth.2404.

13. Bakke, I., Berg, L., Aune, T.E., Brautaset, T., Sletta, H., Tondervik, A., and Valla, S. (2009). Random mutagenesis of the *Pm* promoter as a powerful strategy for improvement of recombinant-gene expression. Appl. Environ. Microbiol. *75*, 2002-2011. 10.1128/AEM.02315-08.

14. Zobel, S., Benedetti, I., Eisenbach, L., de Lorenzo, V., Wierckx, N.J.P., and Blank, L.M. (2015). Tn*7*-Based device for calibrated heterologous gene expression in *Pseudomonas putida*. ACS Synth. Biol. *4*, 1341-1351. 10.1021/acssynbio.5b00058.

15. Caspi, R., Billington, R., Fulcher, C.A., Keseler, I.M., Kothari, A., Krummenacker, M., Latendresse, M., Midford, P.E., Ong, Q., Ong, W.K., et al. (2018). The MetaCyc database of metabolic pathways and enzymes. Nucleic Acids Res. *46*, D633-D639. 10.1093/nar/gkx935.
